# VIPER: variability-preserving imputation for accurate gene expression recovery in single-cell RNA sequencing studies

**DOI:** 10.1186/s13059-018-1575-1

**Published:** 2018-11-12

**Authors:** Mengjie Chen, Xiang Zhou

**Affiliations:** 1 Department of Medicine, University of Chicago, Chicago, IL 60637 USA; 2 Department of Human Genetics, University of Chicago, Chicago, IL 60637 USA; 3 Department of Biostatistics, University of Michigan, Ann Arbor, MI 48109 USA; 4 Center for Statistical Genetics, University of Michigan, Ann Arbor, MI 48109 USA

## Abstract

We develop a method, VIPER, to impute the zero values in single-cell RNA sequencing studies to facilitate accurate transcriptome quantification at the single-cell level. VIPER is based on nonnegative sparse regression models and is capable of progressively inferring a sparse set of local neighborhood cells that are most predictive of the expression levels of the cell of interest for imputation. A key feature of our method is its ability to preserve gene expression variability across cells after imputation. We illustrate the advantages of our method through several well-designed real data-based analytical experiments.

**Electronic supplementary material:**

The online version of this article (10.1186/s13059-018-1575-1) contains supplementary material, which is available to authorized users.

## Introduction

Single-cell RNA sequencing (scRNAseq) technique is becoming increasingly popular in transcriptome studies [1–5]. While previous bulk RNAseq measures average gene expression levels across cells by ignoring potential cell-to-cell heterogeneity, scRNAseq provides an unbiased characterization of gene expression at each single-cell level. The high resolution of scRNAseq has thus far transformed many areas of genomics. For example, scRNAseq has been applied to classify novel cell subtypes [6, 7] and cellular states [2, 4], quantify progressive gene expression [8–12], perform spatial mapping [13, 14], identify differentially expressed genes [15–17], and investigate the genetic basis of gene expression variation [18, 19].

While scRNAseq holds great promise in studies with complex cellular compositions, it also suffers from several important technical disadvantages that limit its use in many settings. These disadvantages include low transcript capture efficiency, low sequencing depth per cell, and wide-spread dropout events, to name a few [20–23]. As a consequence, the gene expression measurements obtained in scRNAseq often contain a large amount of zero values, many of which are due to dropout events [20–23]. For example, a typical drop-seq scRNAseq data can contain up to 90% zero values in the expression matrix [24, 25]. Excess of zero values hinders the application of scRNAseq in accurate quantitative analysis [24–27]. In addition, standard analytic methods developed under bulk RNAseq settings do not account for the excess of zero values observed in scRNAseq data; thus, direct application of these bulk RNAseq methods to scRNAseq often results in sub-optimal performance [20, 28–30].

Several imputation methods have been recently proposed to address the challenges resulted from excess zero values in scRNAseq [24–27]. ScRNAseq imputation relies on the fact that similar cells or correlated genes often contain valuable information for predicting the missing value of a given gene in a given cell. By borrowing information across other cells or other genes, scRNAseq imputation methods construct predictive models to fill in the missing expression measurements. For example, the imputation method SAVER borrows information across genes that are correlated with the gene of interest and uses penalized regression models to impute its missing values [24]. MAGIC constructs a power transformed cell-to-cell similarity matrix and borrows information across cells that are similar to the cell of interest for imputation [25]. scImpute first clusters cells into different subpopulation and then uses only cells within the same subpopulation to perform imputation [26]. Finally, DrImpute clusters cells into different subpopulations, uses each subpopulation in turn to predict the expression level for the cell of interest, and eventually averages these predicted values across all subpopulations as the final imputed value [27].

While existing imputation methods have yielded promising results, they also have important drawbacks. For example, methods such as MAGIC perform imputation based on a low-dimensional space projected from the data, but imputation on a low-dimensional space will likely eliminate gene expression variability across cells and thus abolish a key feature of single-cell sequencing data [25, 26]. As another example, some methods treat all zero expression values as missing data, but failing to distinguish a zero that is due to dropout event from low expression may lead to a loss in imputation accuracy [26, 27]. In addition, some existing imputation methods rely on algorithms that require input parameters that are difficult and even impossible to pre-specify in real data applications. For example, methods such as scImpute require knowing the true number of cell subpopulations in the data a priori, and sometimes also the number of low-dimensional factors that are used to classify these cell subpopulations [26, 27]. As we will show later, misspecification of the number of cell subpopulations in these methods can introduce artificial clusters to imputed data set. In contrast, some method such as SAVER relies on a Markov chain Monte Carlo algorithm to infer all tuning parameters in a sophisticated model, but by inferring all tuning parameters, it becomes extremely slow computationally and may not be applicable to large data sets [24].

Here, we describe a straightforward, accurate, free-of-tuning, and relatively computationally efficient scRNAseq imputation method, which we refer as the Variability-Preserving ImPutation for Expression Recovery (VIPER). VIPER borrows information across cells of similar expression pattern to impute the expression measurements in the cell of interest. However, unlike some of the previous cell-based imputation methods, VIPER does not perform cell clustering before imputation nor uses only cells that belong to the same cell subpopulation for imputation. Instead, VIPER applies a sparse nongenerative regression model to actively select a sparse set of local neighborhood cells that are most predictive of the cell of interest. The selection of this sparse set of cells is done in a progressive manner, and their associated imputation weights are estimated in the final estimation step to ensure both robustness and computational scalability. In addition, VIPER explicitly accounts for expression measurement uncertainty of the zero values in scRNAseq by modeling the dropout probability in a cell-type-specific and gene-specific fashion. VIPER uses an efficient quadratic programing algorithm that infers all modeling parameters from the data at hand while keeping computational cost in check. A key feature of VIPER is its ability to preserve gene expression variability across cells after imputation. We apply our method and compare it with existing imputation methods in several real scRNAseq data-based analytical experiments. We show that, compared to existing methods, VIPER achieves better imputation accuracy, preserves gene expression variability across cells, recovers gene expression measurements that better resemble the bulk RNAseq measurements in the same cell type, and facilitates more reproducible differential expression analysis.

## Materials and methods

### Imputation model and parameter estimation

We aim to impute the zero values in the gene expression matrix of scRNAseq by borrowing information across cells. To do so, we denote *C*_*i*, *j*_ as the observed gene expression count for *i*th cell and *j*th gene, with *i* ∈ {1, ⋯, *n*} and *j* ∈ {1, ⋯, *m*}. We denote ${N}_i={\sum}_{j=1}^m{C}_{i,j}$ as the total read depth for *i*th cell and obtained normalized gene expression levels in terms of RPM (reads per million reads) defined as ${R}_{i,j}=\frac{C_{i,j}}{N_i}\times {10}^6$. While we use RPM in the present study, we note that our method is not restricted to the units of measurement and is applicable to alternative normalized measurements such as TPM (transcripts per kilobase per millions reads) or RPKM (reads per kilobase per millions reads). We denote *X*_*i*, *j*_ as the normalized expression level obtained from RPM values by further performing a log transformation *X*_*i*, *j*_ = log(*R*_*i*, *j*_ + 0.1). For imputation, we examine one cell at a time. For *i*th cell, we assume that its normalized expression level for the *j*th gene in expectation can be expressed as a summation of the expression levels of the same gene across all other cells

$$ E\left({X}_{i,j}\right)={\sum}_{l\in \left\{1,\cdots, i-1,i+1,\cdots, n\right\}}{X}_{l,j}{b}_{i,l}, $$

where *b*_*i*, *l*_ is the predictive effect of *l*th cell on *i*th cell. Note that we only specify a mean model as we only intend to perform single imputation. Single imputation of the mean does not require a full modeling specification for the response variable *X*_*i*, *j*_ (more details in the “Discussion” section).

We assume that the predictive effects *b*_*i*, *l*_ are all nonnegative with ∑_*l* ∈ {1, ⋯, *i* − 1, *i* + 1, ⋯, *n*}_*b*_*i*, *l*_ = 1, so that all *b*_*i*, *l*_ are bounded between 0 and 1 and can be naturally interpreted as imputation weights. Besides the ease of interpretation, bounding all *b*_*i*, *l*_ also ensures imputation stability. Under the above model, the expression levels of *i*th cell are represented as a weighted summation of the expression levels of all other cells. In practice, we would expect only a small set of cells to be informative for imputing the expression levels for the cell of interest. Therefore, we set the estimated small weights to be exactly zero if they are below a certain threshold of *t* = 0.001 (i.e., hard thresholding). Thresholding weights allows us to identify a small set of neighborhood cells for imputation. With hard thresholding and nonnegative weight constraint, our model becomes effectively a sparse nonnegative regression model [31]. Note that, because we model each cell separately, the neighborhood cell list is not symmetric: the fact that *l*th cell is a neighborhood of *i*th cell does not guarantee that *i*th cell is also a neighborhood of *l*th cell. This asymmetric pattern ensures that the identification of neighborhood cells is optimal for each cell. Once we obtain the parameter estimates ${\widehat{b}}_{i,l}$, for each missing data *X*_*i*, *j*_ in turn, we then plug in these estimates to obtain a predicted value ${\widehat{X}}_{i,j}={\sum}_{l\in \left\{1,\cdots, i-1,i+1,\cdots, n\right\}}{X}_{l,j}{\widehat{b}}_{i,l}$ as the imputed value. To reduce the influence of missing values in the weight estimation, the model is fitted using genes that have a zero rate less than a threshold (set to be 10% in the analyses presented in this paper).

We estimate the predictive effect parameters *b*_*i*, *l*_ from the above mean model using ordinary least squares with a quadratic programming algorithm. Specifically, we re-formulate parameter estimation problem in the above model to an optimization problem, where, for each cell *i* in turn, we aim to obtain a set of *i*th cell-specific prediction weights (*b*_*i*, 1_, ⋯, *b*_*i*, *i* − 1_, *b*_*i*, *i* + 1_, ⋯, *b*_*i*, *n*_) from all other cells by minimizing the sum of square prediction errors for

$$ \underset{\left\{{b}_{i,1},\cdots, {b}_{i,i-1},{b}_{i,i+1},\cdots, {b}_{i,n}\right\}}{\min }{\sum}_{j=1}^m{\left({X}_{i,j}-{\sum}_{l\in \left\{1,\cdots, i-1,i+1,\cdots, n\right\}}{X}_{l,j}{b}_{i,l}\right)}^2, $$

where the nonnegative effects *b*_*i*, *l*_ satisfy the constraint that *b*_*i*, 1_ + ⋯ + *b*_*i*, *i* − 1_ + *b*_*i*, *i* + 1_ + ⋯ + *b*_*i*, *n*_ = 1, with the non-zero effects being above a certain threshold of *t* = 0.001. To optimize the above function, we denote *W*_*i*, *j*_ = *X*_*i*, *j*_ − *X*_*n*, *j*_, *Y*_*l*, *j*_ = *X*_*l*, *j*_ − *X*_*n*, *j*_, and *β* = (*b*_*i*, 1_, ⋯, *b*_*i*, *i* − 1_, *b*_*i*, *i* + 1_, ⋯, *b*_*i*, *n*_)^*T*^. The above constrained optimization problem can be re-expressed as a quadratic programming problem

$$ \underset{\upbeta}{\min}\frac{1}{2}{\beta}^T\left({\sum}_{j=1}^m{Y}_{.j}{Y}_{.j}^T\right)\beta -\left({\sum}_{j=1}^m{W}_{i,j}{Y}_{.j}^T\right)\beta, $$

subject to $$ \left(\begin{array}{c}{I}_{\left(n-1\right)\times \left(n-1\right)}\\ {}-{I}_{\left(n-1\right)\times \left(n-1\right)}\end{array}\right)\beta \le \left(\begin{array}{c}{1}_{\left(n-1\right)}\\ {}{0}_{\left(n-1\right)}\end{array}\right) $$, where *I*_(*n* − 1) × (*n* − 1)_ denotes a (*n −* 1) by (*n −* 1) identity matrix, 1_(*n* − 1)_ denotes a (*n −* 1)-vector of 1s, and 0_(*n* − 1)_ denotes a (*n* − 1)-vector of 0s. We solve the optimization problem using quadratic programming [32]. Once we obtain the prediction weight estimates, we further set those weights less than the threshold of *t* = 0.001 to be exactly zero to ensure sparsity. Finally, we re-normalize the non-zero weights to ensure a summation of one.

While the above quadratic programming algorithm is effective, we find that the algorithm is computationally inefficient and does not scale well to large-scale scRNAseq data sets. To ensure algorithm scalability and avoid model overfitting, we perform a pre-selection procedure to first select a small set of candidate cells that will be eventually selected as local neighbors. Specifically, for each cell *i* in turn, we apply standard penalized regression model (lasso or elastic net, with the default tenfold cross validation to determine the penalty parameter) using a random sampled set of 5,000 genes to identify a set of candidate cells that are predictive of the expression of the *i*th cell. Among these candidate cells, we apply the quadratic programming algorithm described previously to further identify a set of neighborhood cells for final imputation. Therefore, our imputation method eventually consists of two steps: a lasso/elastic net-based pre-selection step and a quadratic programming algorithm-based fine tuning and estimation step. With two separate steps, our method ensures computational scalability while avoiding model overfitting by sequentially reducing model complexity.

Finally, we note that a zero count can be generated by two possible mechanisms: it either comes from a dropout event or represents a low or zero level of gene expression. If the zero value of *C*_*l*, *j*_ is due to a dropout event, then it is not an accurate measurement of the true expression level of *j*th gene in *l*th cell. Subsequently, we do not wish to use the normalized value *X*_*l*, *j*_ from a dropout event to impute *X*_*i*, *j*_. However, if the zero value of *C*_*l*, *j*_ comes from low or zero expression level of *j*th gene in *l*th cell, then we would want to use the normalized value *X*_*l*, *j*_ to impute *X*_*i*, *j*_. To distinguish between these two possibilities, we estimate an expected expression level for any zero value of *C*_*l*, *j*_ and use these estimates to perform imputation. The modeling and estimation details for this dropout adjustment step are provided in detail in the Additional file 1: Supplementary Text. Briefly, we assume that the gene expression levels of the *j*th gene for all selected neighborhood cells for the *i*th cell of interest follow a zero-inflated Poisson mixed model, such that *C*_*l*, *j*_~*p*_*i*, *j*_*δ*_0_ + (1 − *p*_*i*, *j*_)*PMM*(*N*_*l*_*λ*_*l*, *j*_, *ψ*_*i*, *j*_). In the model, *p*_*i*, *j*_ represents the dropout probability of *j*th gene that is specific for all neighborhood cells of the *i*th cell of interest; *δ*_0_ denotes a point mass at zero; *N*_*l*_ is the total read depth for the *l*th cell; *λ*_*l*, *j*_ is the Poisson rate parameter that represents the expression level of *j*th gene in the *l*th cell; *ψ*_*i*, *j*_ is an over-dispersion parameter that is specific for *j*th gene and for all neighborhood cells of the *i*th cell of interest; and PMM denotes a Poisson mixed effects model. Under the zero-inflated Poisson mixed effects model, *C*_*l*, *j*_ is exactly zero with a dropout probability *p*_*i*, *j*_ and follows an over-dispersed Poisson distribution with probability 1 − *p*_*i*, *j*_. Our goal is to estimate *λ*_*l*, *j*_, the underlying expression level of *j*th gene in *l*th cell, to serve as our final predictor variable for all zero values of *C*_*l*, *j*_. To do so, we first estimate all parameters (i.e., *p*_*i*, *j*_, *λ*_*l*, *j*_, *ψ*_*i*, *j*_) through an expectation maximization (EM) algorithm based on the selected neighborhood cells for the *i*th cell of interest. Afterwards, we obtain an estimate of ${\widehat{\lambda}}_{l,j}$ and use it to replace *X*_*l*, *j*_ to serve as the final predictor variable. Certainly, we use the normalized expression measurement *X*_*l*, *j*_ directly as the predictor variable if *C*_*l*, *j*_ is non-zero.

We refer to our method described above as the Variability-Preserving ImPutation for Expression Recovery (VIPER). We note that the non-statistical term “variability preserving” in the method name refers to the fact that our method is capable of preserving gene expression variance across cells after imputation, as we will show in the following real data-based analytic experiments. The property of “variability preserving” in our method contrasts a few other imputation methods that aggressively reduce variance across cells after imputation (e.g., MAGIC and scImpute). However, we also acknowledge that, just like any existing single-cell imputation method, our method is a single imputation method that suffers from the usual drawbacks when compared to other more advanced imputation methods such as multiple imputation [33].

### Real data sets

We examine four scRNAseq data collected from three studies. These three studies include both unique molecular identifier (UMI)-based techniques (CEL-seq; the first study) and non-UMI-based techniques (Fluidigm C1; the second two studies).

Specifically, the first data set is from Grun et al. [34]. It is a mouse study that examines a total of 251 cells that were cultured in two different media. These cells include 74 embryonic stem cells (ESCs) cultured in a two-inhibitor (2i) medium, 45 ESCs cultured in a serum medium, 56 samples with pooled RNA from ESCs cultured in a 2i medium, and 76 samples with pooled RNA from ESCs cultured in serum. The first two types of cells are single-cell measurements while the second two types of samples are measurements averaged across single cells. We obtained raw UMI count measurements for 23,459 genes from the authors. We selected genes that are expressed in at least 10% of the cells and analyzed a total of 12,184 genes in the final analyses.

The second and third data are both from Chu et al. [35]. The second data set contains gene expression measurements for 1,018 single cells from both human ESCs and the lineage-specific progenitor cells derived from these ESCs. We refer to the second data as the “Cell Type” data because the cells belong to seven known cell subpopulations that include neuronal progenitor cells (NPCs), definitive endoderm derivative cells (DEDs), endothelial cells (ECs), trophoblast-like cells (TBs), undifferentiated H1 and H9 ESCs, and foreskin fibroblasts (HFFs). Note that above we denoted the definitive endoderm derivative cells as DEDs instead of the traditional notation of DEs because we later used DE to represent differential expression. Besides the single-cell data, the second data also contains 19 corresponding samples from bulk RNAseq. The third data set contains gene expression measurements for 758 single cells. We refer to the third data set as the “Time Course” data as the cells are collected at six different time points (0, 12, 24, 36, 72, and 96 h) during the developmental trajectory of ESCs differentiating towards DEDs. In addition to the single-cell data, the third data also contains 15 samples from bulk RNAseq for all other time points except for 0 h. We downloaded both the second and third data in terms of four expected count matrices from the Gene Expression Omnibus (GEO) website with accession number GSE75748. We filtered out genes that are expressed in less than 10% of the cells and analyzed a total of 13,829 genes in the Cell Type data and 13,059 genes in the Time Course data.

The fourth data is from Shalek et al. [15]. It contains gene expression measurements for 1,700 primary mouse dendritic cells (DCs) stimulated with three pathogenic components for different amount of time (1 h, 2 h, 4 h, and 6 h). The three pathogenic components include lipopolysaccharide (LPS) which is a component of Gram-negative bacteria, PAM3CSK4 (PAM) which is a synthetic mimic of bacterial lipopeptides, and PIC which is a viral-like double-stranded RNA. For this data, we retained cells with library sizes greater than one million. After further filtering out genes that are expressed in less than 10% of the cells, we focus on a final set of 1,053 cells with 16,702 genes for the following analysis.

### Real data-based experiments

We performed three different experiments using published scRNAseq data. Two experiments (masking and comparison to bulk RNAseq) are described in detail in the “Results” section. We describe the details of the third down-sampling experiment in the following paragraphs. The down-sampling experiment is performed on one real data at a time and consists of two steps.

In the first step, for each gene in turn, we randomly sampled gene expression values across all cells based on a multinomial distribution. In this multinomial distribution, the cell-specific probability parameters are set to be the corresponding cell expression proportion in the original data, while the total read count parameter is set so that the down-sampled read depth represents a fixed proportion of the original read depth (i.e., one minus the down-sampling rate, where the down-sampling rate is set to be either 0.5, 0.6, 0.7, 0.8, 0.9, or 0.95; thus, 0.5 represents the setting where the library size is reduced to 50% and thus corresponds to a larger down-sampling rate compared to 0.95). The multinomial distribution ensures that the expected gene expression proportion of each cell remains unchanged after down-sampling.

The down-sampled data from the first step contains both zero values and non-zero values. After the initial down-sampling step, we further introduce extra dropout events to the non-zero values as a second step of the down-sampling experiment, in order to mimic data generating process of real scRNAseq data. These dropout events are introduced to each of the non-zero values by sampling from a Bernoulli distribution characterized by a dropout rate. The dropout rate is designed using two different strategies. In the first strategy, we set a fixed dropout rate of 0.8 that is independent of the non-zero value from the initial multinomial sampling step. In the second strategy, we model the dropout rate as a logistic function of the non-zero down-sampled value. This logistic function is estimated in the original data for each cell subpopulation separately. Specifically, within each cell subpopulation, for each gene in turn, we obtained the percentage of zero values and the mean of non-zero values across all cells. Treating the zero percentage as outcome and the non-zero means as explanatory variable, we fitted a logistic regression model to establish their quantitative relationship in each cell subpopulation. Afterwards, we used the fitted logistic model to compute a dropout rate for each non-zero value in the data obtained from the initial down-sampling step. With either strategy, we set each non-zero value to be zero with a probability equal to the dropout rate. Therefore, the zero values in the final down-sampled data are either due to low expression values in the original data and the subsequent multinomial down-sampling (i.e., first step) or due to the extra dropout events (i.e., second step).

We examined several down-sampling scenarios based on various down-sampling rates. In each scenario, we performed imputation on the zero values resulting from either multinomial down-sampling or from the additional dropout events. We measured imputation accuracy by comparing the imputed data to the original data across all entries. Note that the zero values that were already present in the original data were not analyzed here because we do not know their “true” values in the original data.

### Methods for comparison

We compared our method with four existing imputation methods. These imputation methods include DrImpute [27] (version 1.0), MAGIC [25] (version 0.1.0), SAVER [24] (version 0.3.1), and scImpute [26] (version 0.0.5). Note that we downloaded most of these software versions and compared them in 2017 when the original papers describing these methods were unpublished. We carried out analyses following the recommended procedure from each software. The software scImpute also requires a specification of the number of cell subpopulations, which is generally unknown in most real data. Here, we set the number of cell subpopulations required by scImpute to be the truth in all our experiments (except those described in the “Discussion” section). To assess imputation accuracy, in one experiment, we performed differential expression analysis between pairs of cell subpopulations following imputation. For differential expression analysis, we used three different DE software that include DEseq2 [36] (version 1.16.1), edgeR [37–39] (version 3.20.7; with either the likelihood ratio test, LRT, or with the quasi-likelihood *F* test, QLF), and SCDE [20] (version 1.99.1). Note that SCDE was specifically designed for single-cell DE analysis. Both edgeR and DEseq2 were originally designed for bulk RNAseq studies and do not account for the excessive zero values encountered in the single-cell RNAseq data. However, recent comparative studies have suggested that the QLF version of edgeR and DESeq2 enjoy superior performance for single-cell DE analysis than many single-cell data-specific DE methods [38]. We carried out differential expression analyses also following the recommended procedure from each software.

## Results

### Method and analysis overview

**Fig. 1:**
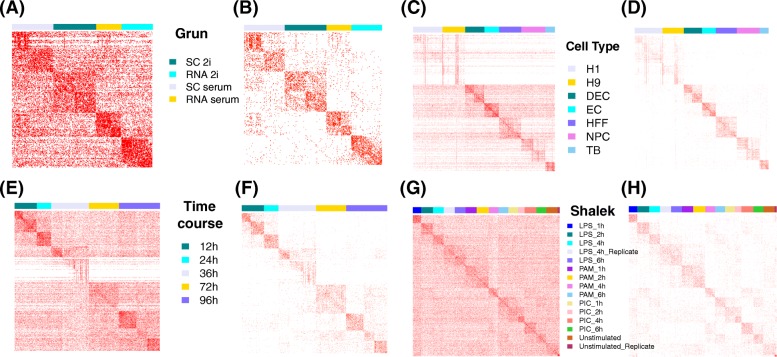
Estimated imputation weights inferred in the two steps of the VIPER method in four data sets. Results are shown for Grun data (**a**, **b**), Cell Type data (**c**, **d**), Time Course data (**e**, **f**), and Shalek data (**g**, **h**). Cells with non-zero weights are shown in red. Color labels on top of each heatmap represent different cell subpopulations in that data set. Compared to the pre-selection step (**a**, **c**, **e**, **g**), the sparsity of non-zero weights further reduced after the estimation step (**b**, **d**, **f**, **h**) in all data sets

The technical details of VIPER are described in the “Materials and methods” section with an illustration of the imputation procedure provided in Additional file 2: Figure S1. Briefly, VIPER examines one cell at a time, searches for a small subset of neighborhood cells that are predictive of its gene expression levels, and finally imputes its missing expression measurements using its neighborhood cells. For imputation, VIPER relies a sparse nonnegative regression model to model the gene expression levels of the cell of interest as a weighted summation of a sparse set of its neighborhood cells. These neighborhood cells and their imputation weights are inferred through a computationally efficient two-step procedure that includes a pre-selection step and an estimation step. In the pre-selection step, VIPER identifies a moderate-sized set of candidate cells that are likely predictive of the expression levels of the cell of interest and that will serve as a candidate pool for the final selection of neighborhood cells. The pre-selection step is done efficiently using a standard penalized regression method based on either lasso or elastic net and is designed to mitigate the computation burden of the later estimation step. In the estimation step, with the selected candidate cells, VIPER fits a sparse nonnegative regression model using a quadratic programming algorithm to further identify a final set of neighborhood cells and estimate their weights for imputation. The size of the final set is often a few times smaller than the candidate pool (Fig. 1). As a consequence, VIPER reduces model complexity in a sequential fashion, which can help to avoid overfitting. Finally, when imputing the zero values, VIPER also explicitly models a gene-cell-specific dropout probability to distinguish between zero due to a dropout event and due to a low or zero level of gene expression, which further improves imputation accuracy.

We use four real data sets from three published studies to evaluate the performance of our method. The details of these data are described in the “Materials and methods” section. We refer to the first data as the Grun et al. data [34], the second data as the Cell Type data which is obtained from Chu et al. [35], the third data as the Time Course data which is also obtained from Chu et al. [35], and the fourth data as Shalek et al. data [15]. Both the Cell Type and Time Course data also contain a corresponding bulk RNAseq data. We used all data sets in most of our evaluation experiments described in the following sections. In addition, we used primarily the Cell Type and Time Course data from Chu et al. [35] for some simple illustrations described in the “Discussion” section and for an experiment that requires corresponding bulk RNAseq data.

We compare the performance of our method with existing approaches, including (1) DrImpute, which relies on pre-identified cell subpopulations for imputation [27]; (2) MAGIC, which uses a power-transformed cell similarity matrix constructed using a few number of principal components to perform imputation [25]; (3) SAVER, which uses genes that are correlated with the gene of interest to perform imputation [24]; and (4) scImpute, which also uses pre-identified cell subpopulations to perform imputation [26]. We access the accuracy of different imputation methods by performing four real data-based experiments.

### Assessing imputation accuracy through data masking

**Fig. 2:**
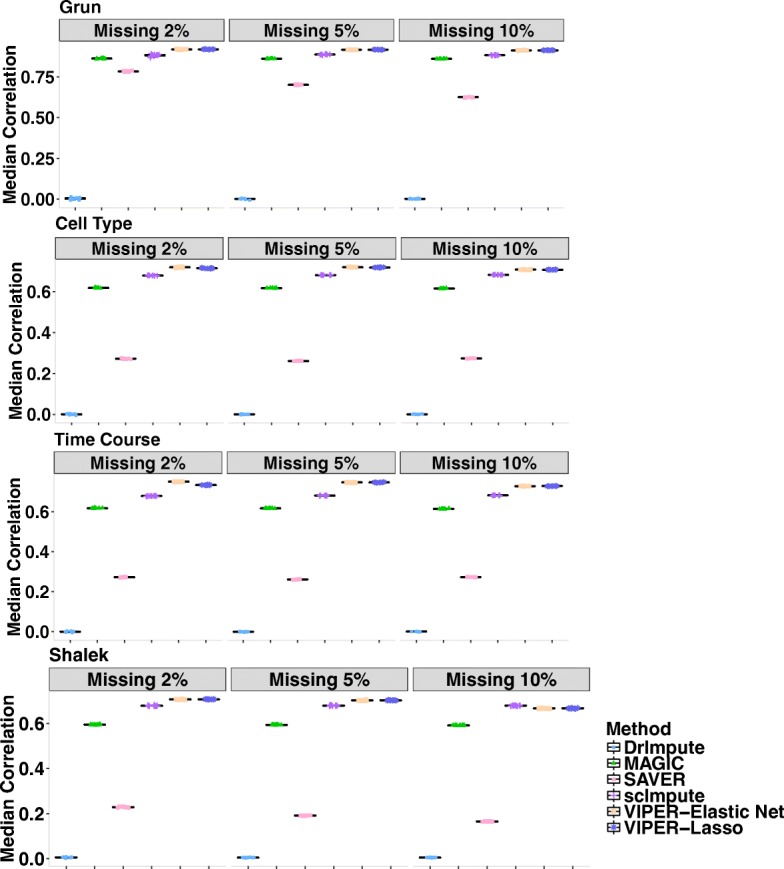
Correlation between the masked truth and imputed values by different methods in the data masking experiment. Rows represent the four different data sets (Grun, Cell Type, and Time Course, Shalek) used in the experiment. Columns represent masking percentage (2%, 5%, and 10%). Methods for comparison include DrImpute (blue), MAGIC (green), SAVER (pink), scImpute (purple), VIPER with elastic net selection (peach), and VIPER with lasso selection (dark blue). Boxplots show correlation values obtained from 10 masking replicates, where in each replicate we calculated the correlation for each cell in turn and plotted the median correlation value across cells

First, we assess imputation performance of different methods in recovering randomly masked non-zero gene expression values. To do so, in each of the four scRNAseq data (Grun, Cell Type, Time Course, Shalek), we randomly selected a fixed percentage (2%, 5%, or 10%) of non-zero entries in the observed data matrix and masked these values to be zero to generate a new gene expression matrix. We then apply different methods to the newly generated gene expression matrix and compute the correlation between the imputed values and the masked values across all entries as a measurement of imputation accuracy. For each data set, we perform 10 masking replicates and plot the results across these replicates in Fig. 2. Overall, VIPER outperforms all other existing approaches in all data sets. The performance of our method is followed by scImpute and MAGIC, while SAVER and DrImpute do not perform well. For example, in Cell Type data, when masking percentage is 2%, the correlation between the imputed values by VIPER and masked truth is 0.71 (when lasso is used in the pre-selection step) or 0.72 (when elastic net is used in the pre-selection step), while the correlation by DrImpute is 0.0005, by MAGIC is 0.62, by SAVER is 0.27, and by scImpute is 0.68. In addition, as one might expect, the performance of our method and the other methods decay slightly with the increasing of masking percentage, though the rankings of different methods remain the same. For example, in Cell Type data, when masking percentage increases to 10%, the correlation between the imputed values by VIPER and the truth is 0.70 (for lasso) or 0.71 (for elastic net), while the correlation by DrImpute is 0.0007, by MAGIC is 0.61, by SAVER is 0.27, and by scImpute is 0.68. The rankings of different methods are similar when we use squared loss (a.k.a mean squared error) and L1 loss (a.k.a. mean absolute deviation) as alternative imputation accuracy measurements (Additional file 2: Figures S2–S4). For example, in Cell Type data, when masking percentage is 2%, the square loss for the imputed values when compared to the masked truth is 0.66 (for lasso) or 0.651 (for elastic net) by VIPER, while the square loss by DrImpute is 15.698, by MAGIC is 3.029, by SAVER is 13.696, and by scImpute is 0.98. The masking experiments suggest that our method is capable of accurately recovering the true expression levels in real data.

### Assessing imputation accuracy through down-sampling

**Fig. 3:**
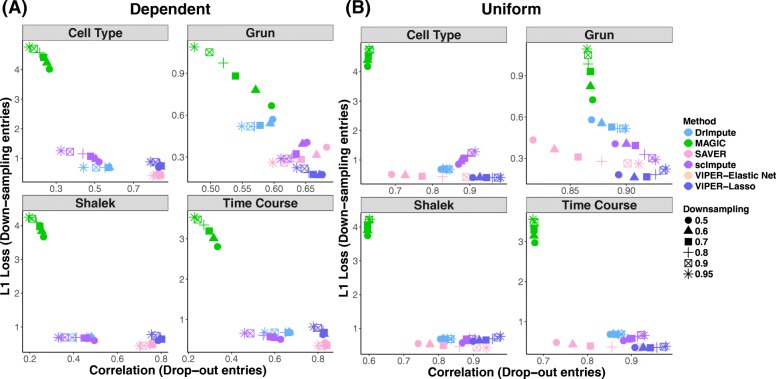
Imputation accuracy in the down-sampling experiment. Results are shown for down-sampling experiments using either expression-dependent dropout rate (**a**) or expression-independent dropout rate (**b**) for four different data sets (Gurn, Cell Type, Time Course, and Shalek). Imputation accuracy are measured by comparing imputed values to the original truth and are evaluated for two different types of zeros separately: zeros that are due to low expression level in the original data and the multinomial subsampling step (down-sampling entries; *y*-axis) and zeros that are due to dropout events (dropout entries; *x*-axis). Accuracy is measured by correlation for the dropout entries and by L1 loss for the down-sampling entries (because of an excess of zero values in the original data for the down-sampling entries). Color of the dots represents methods for comparison: DrImpute (blue), MAGIC (green), SAVER (pink), scImpute (purple), VIPER with elastic net selection (peach), and VIPER with lasso selection (dark blue). Shape of the dots represents the down-sampling rate used in the multinomial subsampling step

Second, we assess the performance of different imputation methods using two down-sampling experiments. The procedure of the down-sampling experiments is described in detail in the “Materials and methods” section with an illustration in Additional file 2: Figure S5. Specifically, we generated down-sampled version of each of the four scRNAseq data through multinomial down-sampling, created additional zero values by introducing dropout events, applied different methods to impute the zero entries in the down-sampled data, and examined whether these imputed values recover the known truth compared to the original data. The dropout events are introduced using a rate that is either dependent or independent on the expression values (details in the “Materials and methods” section). The zero values due to low expression level and the multinomial down-sampling or due to the additional dropout events vary across different data sets (Additional file 3: Table S1). For example, with a dropout rate dependent on the expression values and a down-sampling rate of 0.9, 8.52% of the zero values in the Grun down-sampled data are due to multinomial down-sampling (while 91.48% are due to dropout); 43.7% of zeros in the Cell Type data, 35.59% in the Time Course data, and 29.38% in the Shalek data are due to multinomial down-sampling. The down-sampling experiment provides a unique opportunity for us to assess the performance of different imputation methods for imputing these two different types of zeros separately. To assess imputation accuracy, for zeros due to dropout, we computed the correlation between the imputed data and the original data before down-sampling. For zeros due to low abundance and down-sampling, we calculated the L1 loss between the imputed data and the original data before down-sampling; we did not use correlation for down-sampling zeros here because correlation is no longer an effective measurement due to the excessively large number of zeros in the original data for these down-sampling zeros. The results are shown in Fig. 3. Corresponding results measured with L1 loss for both these two types of dropout rates are consistent with the main results and are shown in Additional file 2: Figure S6.

For the zeros due to dropouts, VIPER outperforms most other imputation approaches across the four different data sets and the two different dropout rate settings: it is ranked as the best method in six out of the eight scenarios examined. The only exceptions are Time Course data as well as the Grun et al. data with an expression-dependent sampling rate (i.e., one scenario out of eight), where, in the later case, while VIPER outperforms SAVER when the down-sampling rate is high (0.6 to 0.95), it performs slightly worse than SAVER in the presence of a low down-sampling rate (0.5). The performance of our method in other settings is generally followed by SAVER and then scImpute, while DrImpute and MAGIC do not work well. For example, in the Cell Type data, when the down-sampling rate is 0.5, the correlation between the imputed values in the down-sampled data and the truth in the original data is 0.830 using lasso and 0.832 using elastic net by VIPER, while the correlation by DrImpute is 0.572, by MAGIC is 0.266, by SAVER is 0.828, and by scImpute is 0.522. The rankings of different methods also remain largely the same when we use different down-sampling rates or alternative down-sampling strategies.

For the zeros due to low expression abundance and multinomial down-sampling, VIPER outperforms all other imputation approaches except for SAVER across the four different data sets and the two different dropout rate settings. VIPER and SAVER were each ranked as the best method in four out of the eight scenarios. In particular, VIPER produces better results than SAVER in three out of four data sets when we use expression-independent dropout rates and produces better results in one out of four data sets when we use expression-dependent dropout rate. The good performance of SAVER in half of the scenarios for imputing zeros due to low expression abundance is presumably due to the fact that the imputed values from SAVER do not differ much from the unimputed data in general (which will become apparent in the next section); thus, the imputed values from SAVER for these zeros due to low expression abundance remain close to zero. The performance of our method and SAVER is followed by either DrImpute or scImpute, depending on the data set, while MAGIC does not work well here. For example, in the Cell Type data, when the down-sampling rate is 0.5, the L1 loss between the imputed values in the down-sampled data and the truth in the original data is 0.405 (for either lasso or elastic net) by VIPER, while the L1 loss by DrImpute is 0.686, by MAGIC is 4.182, by SAVER is 0.514, and by scImpute is 0.857. The rankings of different methods remain largely the same when we use different down-sampling rates.

Therefore, consistent with the masking experiments, the down-sampling experiments here also suggest that VIPER is capable of accurately recovering the true expression levels in real data.

### Assessing imputation accuracy by comparing to bulk RNAseq

**Fig. 4:**
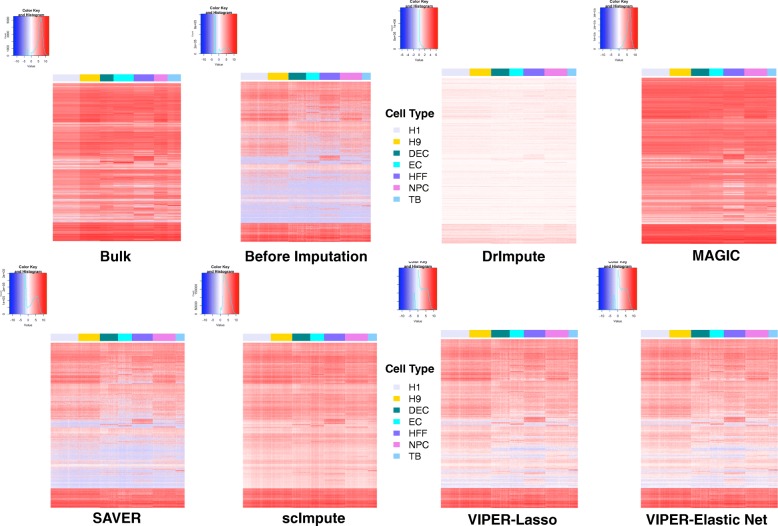
Heatmaps show the unimputed or imputed gene expression measurements in the scRNAseq data together with the gene expression measurements from bulk RNAseq in the Cell Type data. Expression measurements are shown across cells (for scRNAseq) or across sample replicates (for bulk RNAseq) in seven different cell subpopulations. The seven different cell subpopulations include H1, H9, DEC, EC, HFF, NPC, and TB. Imputed scRNAseq data are obtained from different imputation methods that include DrImpute, MAGIC, SAVER, scImpute, VIPER with elastic net selection, and VIPER with lasso selection

Third, we assess the performance of different methods by comparing the imputed gene expression values from scRNAseq to the expression values measured by bulk RNAseq in the same cell subpopulation. To do so, we rely on the Cell Type and Time Course data from Chu et al. [35] that also have bulk RNAseq data measured in the same cell subpopulations. We apply different methods to perform imputation in each data and display the imputed values from scRNAseq together with the bulk RNAseq data in Fig. 4 (for Cell Type data) and Additional file 2: Figure S7 (for Time Course data). The gene expression heatmaps show that there are almost no zero entries in the bulk RNAseq data, but there is a large proportion of zero entries in the raw scRNAseq data before imputation. In addition, scRNAseq data display a substantial gene expression variation across cells within each cell subpopulation. Intuitively, a good imputation method would generate an expression heatmap lying somewhere between the bulk RNAseq data and the raw scRNAseq data: the imputed data should contain mean gene expression levels that are consistent with the bulk RNAseq data, but also maintain substantial gene expression variation across cells within the subpopulation. After imputation, with the exception of SAVER (and to a less extent of DrImpute), we found that most imputation methods are able to replace a large proportion of zero entries with imputed values. In addition, the imputed data from VIPER (with lasso or elastic net) lies somewhere between the bulk RNAseq data and the raw unimputed scRNAseq data, with reasonably accurate mean estimation and substantial variation across cells. In contrast, the imputed data from SAVER looks very similar to the raw scRNAseq data before imputation, while the imputed data from scImpute and MAGIC only resemble the bulk RNAseq data well. Careful examination of the imputed data heatmap also suggests that the imputed data from DrImpute appears to be deprived much of the gene expression variation across genes, while the imputed data from MAGIC (and to a less extent scImpute) appears to be deprived much of the gene expression variation across cells—even though the mean imputed gene expression levels across cells within each cell subpopulation from MAGIC resemble quite closely to that of bulk RNAseq.

**Fig. 5:**
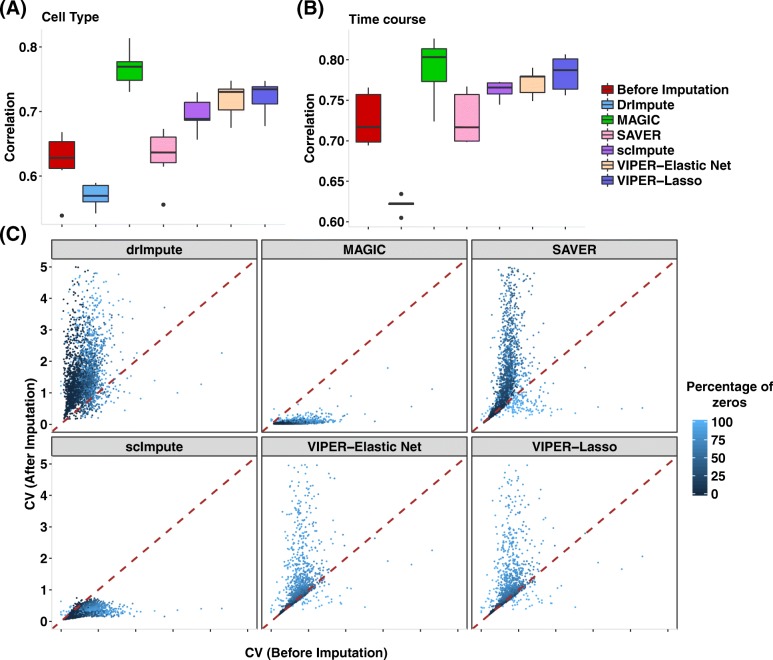
Quantifying imputed gene expression in scRNAseq. **a**, **b** Correlation between imputed scRNAseq data and bulk RNAseq data across different cell subpopulations in the Cell Type data (**a**) and the Time Course data (**b**). Correlation is computed between the mean gene expression measurements averaged across cells with a cell type from scRNAseq and the mean gene expression measurements averaged across sample replicates from bulk RNAseq in the same cell type. For scRNAseq, results are shown for unimputed data (red) and imputed data by different methods that include DrImpute (blue), MAGIC (green), SAVER (pink), scImpute (purple), VIPER with elastic net selection (peach), and VIPER with lasso selection (dark blue). **c** Gene expression variation across cells in imputed scRNAseq data versus that in the raw data for DEC cells in Cell Type data set. For each gene in turn, the coefficient of variation (CV) across all cells after imputation (*y*-axis) is computed and plotted against the CV of non-zero cells before imputation (*x*-axis) for different methods. Each dot represents a gene, and the color of the dot represents the mean of non-zero values

To quantify the performance of different imputation methods in terms of recovering the mean expression level of each cell type, we averaged the expression levels across all cells within a cell subpopulation to obtain an averaged gene expression measurement in the imputed scRNAseq data. For each cell subpopulation in turn, we then computed the correlation between bulk RNAseq and the imputed mean scRNAseq measurements across all genes (Fig. 5a, b). Among these methods, MAGIC produces the highest correlation, suggesting that MAGIC is capable of imputing the mean expression level across cells within each cell subpopulation relatively accurately, even though it eliminates the majority of the variability across cells (see also the next paragraph). In contrast, our method works well in recovering the mean expression level of a cell type while maintaining the expression variability across cells within the same cell type. The performance of our method is followed by scImpute. On the other hand, the imputed mean expression values by SAVER are correlated with bulk RNAseq in the same degree as unimputed data, while the imputed mean expression values by DrImpute are less correlated with bulk RNAseq compared to the unimputed data. The similar or reduced correlation between the imputed data by SAVER/DrImpute and bulk RNAseq data suggests that both SAVER and DrImpute do not improve gene expression measurement accuracy in these data.

To quantify the cross-cell gene expression variability in the imputed data sets, for each gene in turn, we computed the coefficient of variation (CV) across cells after imputation and compared it to the CV of the non-zero values before imputation. We contrasted these two CV values for DEC cells in Cell Type data set and stratified the contrast by showing different zero proportions (Fig. 5c) or different non-zero mean expression levels (Additional file 2: Figure S8) with colors in gradient. Intuitively, for a given gene, if the zero values across cells are all due to dropout events, then we would expect the CV after imputation to be similar to the CV before imputation—because the imputed data would follow the same distribution as the non-zero values before imputation. In contrast, if the zero values are all due to low gene expression levels, then we would expect the CV after imputation to be higher than the CV before imputation—because the imputed data would generally have lower values than the non-zero values before imputation. Therefore, CV after imputation by a proper method would be either equal to or higher than the CV before imputation. Indeed, our method (lasso or elastic net) produces results that meet this expectation, with some genes having similar CV values after imputation while some genes having higher CV values after imputation. In contrast, almost all genes have smaller CV values after imputation by MAGIC (or, to a less extent, by scImpute), suggesting that MAGIC and scImpute reduce gene expression variability across cells after imputation. The reduced variability by MAGIC or scImpute is consistent with the heatmaps shown in Fig. 4. On the other hand, most genes have higher CV values after imputation by either DrImpute or SAVER, suggesting that DrImpute/SAVER effectively treats most zero values as non-dropout events.

Importantly, CV plots in the other three data sets (Time Course, Grun data, and Shalek data) display similar patterns (Additional file 2: Figures S9–S11). We also examined CV plots in the down-sampled data, which allows us to visualize the imputed value variance for two different types of zeros separately: zeros that are due to true zero or low abundance in the original data, and zeros that are due to dropouts. To do so, in the down-sampling experiment, we computed CV for the imputed zeros originating from dropout together with unimputed data and contrast it with the CV from the corresponding original truth (Additional file 2: Figure S12). We also computed CV for the imputed zeros originating from true zero or low abundance together with unimputed data and contrast it again with the CV from the corresponding original truth (Additional file 2: Figure S13). Both CV plots are consistent with our main results, suggesting that our method is capable of preserving variability regardless which type of zeros we focus on.

Overall, both the correlation that measures the expression mean of a cell type (Fig. 5a, b) and the CV that quantifies the expression variability across cells within the cell type (Fig. 5c) suggest that only our method can produce accurate expression measurements while maintaining desired gene expression variability across cells.

### Accurate imputation facilitates reproducible differential expression analysis

**Fig. 6:**
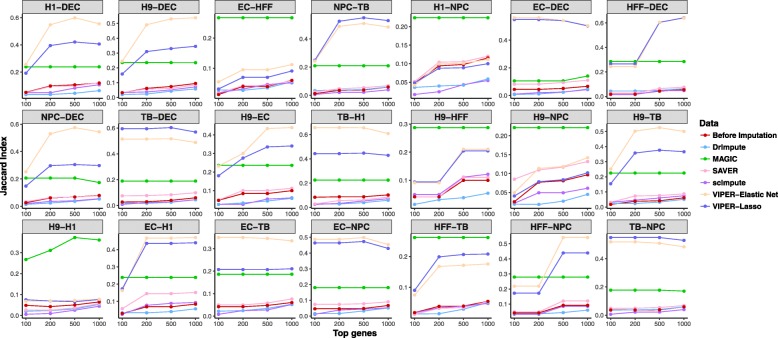
Overlap of top differentially expressed genes identified by SCDE between two data splits of the raw data or imputed data by different methods. SCDE is applied to detect genes that are differentially expressed between pairs of cell subpopulations in the Cell Type data for all pairs of seven cell types. In each comparison, cells from the two cell types are split randomly into two subsets. Imputation and differential expression analysis methods are applied to each data subset separately. The mean Jaccard index between the top 100, 200, 500, or 1000 differentially expressed genes from two subsets are computed across 10 random data splits for each imputation method as a quantification of imputation accuracy, where the Jaccard index is computed as the ratio of the intersection and the union between the top gene lists from the two subsets. Methods for comparison include DrImpute (blue), MAGIC (green), SAVER (pink), scImpute (purple), VIPER with elastic net selection (peach), and VIPER with lasso selection (dark blue)

Finally, we performed differential expression analysis on the imputed data to illustrate the benefits of imputation. Specifically, we focus on detecting differentially expressed genes between pairs of cell subpopulations from the Cell Type data, for all 21 pairs of 7 cell types. For each pair in turn, we randomly split cells into two subsets and applied different DE methods (DESeq2, two different versions of edgeR, and SCDE) to analyze each subset separately. We then computed the proportion of overlap between the top 100, 200, 500, or 1000 DE genes detected from each subset—and we compute this proportion as Jaccard index, defined as the ratio of the intersection and the union between the top gene lists from the two subsets. We performed the random data split 10 times and show the mean overlap proportions of DE genes detected by SCDE from these replicates in Fig. 6. The results based on the DE methods edgeR and DESeq2 are similar and are shown in Additional file 2: Figures S14–S16. We also display the overlap proportions among the top 100 DE genes across replicates for three exemplary pairs (H1 vs DEC, EC vs HFF, and NPC vs TB) in Additional file 2: Figure S17. Intuitively, if an imputation method works well, then DE analysis on the imputed data from the method would yield reproducible results, leading to a high overlap proportion among the top DE genes detected from the two split subsets. Consistent with the higher imputation accuracy of our method observed in the previous experiments, our method indeed achieves consistent DE results between the two data subsets, often a few times more so than the other imputation methods, for most cell type pairs. The performance of our method is often followed by MAGIC, and sometimes SAVER. In contrast, DrImpute (and occasionally scImpute) achieves a lower overlap proportion compared with the unimputed data, again consistent with its low performance in other experiments. As a concrete example, comparing H1 vs DEC, the mean Jaccard index between the top 100 DE genes detected by SCDE from the two data splits is 0.192 (for lasso) or 0.256 (for elastic net) by VIPER, while the overlap proportion is 0.035 by DrImpute, 0.238 by MAGIC, 0.048 by SAVER, 0.047 by scImpute, and 0.05 without imputation. Similarly, the mean Jaccard index between the top 500 DE genes detected from the two data splits is 0.421 (for lasso) or 0.600 (for elastic net) by VIPER, while the Jaccard index is 0.044 by DrImpute, 0.238 by MAGIC, 0.099 by SAVER, 0.082 by scImpute, and 0.106 without imputation. Overall, our method produces reproducible differential expression results between split data sets, suggesting that imputation can facilitate the detection of DE genes.

One thing we noticed in our analysis is that many genes are detected as DE in MAGIC imputed data (Additional file 2: Figure S21), which likely originates from the diminished gene expression variation across cells within each cell subpopulation after imputation (Additional file 2: Figures S18–S20); thus, even a small difference in the mean expression level between two cell subpopulations would lead to a detection of differential expression. As another example, besides MAGIC, we found that scImpute also detects a higher number of DE genes than our methods, which is consistent with the reduced gene expression variation across cells in the imputed data from scImpute (Fig. 5b). Therefore, we caution that the number of DE genes is likely highly influenced by gene expression variation across cells after imputation.

To further validate our results, we also performed analyses by permuting data in both subsets and performing overlap analysis based on the permuted data. In particular, we permuted cell type labels but preserved the expression correlation structure across genes and then performed DE analysis. Intuitively, if an imputation method introduces artificial bias to the data in the sense of facilitating the identification of DE genes not based on their true differential expression evidence but based on other properties of the gene, then this method would also lead to a high overlap proportion between the two permuted data subsets. The results (Additional file 2: Figures S22–S23) show that the overlap proportion detected by all methods, except for MAGIC, in the permuted data are very similar and small, suggesting that most imputation methods unlikely introduce artificial bias to the data and that our previous comparison results are valid. On the contrary, MAGIC introduces an excessive number of artificially overlapped DE genes in the permuted data, consistent with the diminished gene expression variation across cells observed in MAGIC imputed data.

Finally, we emphasize that our method is reasonably computationally efficient. We list the imputation time by all methods for the four data sets in Additional file 3: Table S2. The computation time of different methods depends on the number of cell, number of genes, and number of genes that have missing values. Among these methods, MAGIC is the fastest method among all while SAVER is the slowest, and our method is 5–50 times faster than SAVER. Overall, our method is reasonably computationally efficient while producing more accurate results than the other imputation methods.

## Discussion

We have presented a new method for imputing gene expression levels in scRNAseq data. We have compared its performance with other existing scRNAseq imputation methods in four experiments using published scRNAseq data sets. With these real data examples, we show that our method achieves higher imputation accuracy compared with existing methods, preserves expression variability across cells, and facilitates the robust identification of differentially expressed genes between cell subpopulations.

Besides differential expression analysis, several previous studies have also performed clustering analysis on imputed data to identify cell subpopulations. Evaluating the performance of different imputation methods for clustering analysis in real data is challenging because the underlying subpopulation structures are largely unknown. Even for the data set that consists of several distinct cell types, such as the Cell Type data used in this paper, it is unknown whether some cell types are heterogeneous and consist of cell subpopulations within. Therefore, it is difficult to comprehensively evaluate the performance of different imputation methods for clustering in real data. Instead, we present a simple example here to illustrate the behavior of different imputation methods for clustering analysis, using two cell types (H1 and NPC) from the Cell Type data. Specifically, for each cell type, we display cells based on the top two principal components (PCs) extracted from either the raw data or the imputed data by different methods (Additional file 2: Figures S24–S25). Overall, the clustering results from VIPER imputed data, as visualized in the PC plots, are generally consistent with that from raw data, DrImpute imputed data or SAVER imputed data. However, the clustering results obtained from scImpute or MAGIC imputed data are generally different from the raw data and the rest of the methods. Specifically, scImpute requires the specification of the number of cell subpopulations before imputation, which is unknown in any real data, and which, as is shown below, fully determines the number of cell clusters identified in the imputed data. In particular, when we set the number of cell subpopulations to be either 2, 3, or 4 before imputation (2 is the minimum allowed in scImpute), we also detected 2, 3, or 4 cell subpopulations after imputation, respectively. In contrast, MAGIC produces a parabola-shaped curvature in the H1 cells and a circular-shaped curvature in the NPC cells. While not completely impossible, both curvatures by MAGIC are rather unexpected. Therefore, for any imputation method, we would recommend practitioners to carefully examine clustering results both on the raw data and on the imputed data to arrive at a sensible interpretation.

We have primarily focused on selecting neighborhood cells to impute missing data in scRNAseq. In principle, one could borrow information across cells or across genes to impute a missing value in scRNAseq. Indeed, an alternative imputation strategy that has also been applied before (i.e., SAVER) is to select genes that have similar expression levels as the gene of interest to perform imputation. Our method can be easily adapted to use neighborhood genes to impute missing data. However, in our experience, we find that using neighborhood cells is often more accurate than using neighborhood genes to perform imputation. To illustrate the difference in accuracy between these two different strategies, we perform a simple analysis using the Grun et al. and Cell Type data from Chu et al. [35]. Specifically, we fit the standard lasso penalized linear regression model in two different fashions: (1) either predict the expression of a given cell by regressing on the expression of all other cells; (2) or predict the expression of a given gene by regressing on the expression of all other genes. We measure the prediction performance using in-sample *R*^2^ and find that prediction based on cells are much more accurate than prediction based on genes (Additional file 2: Figure S26). Therefore, we have primarily focused on illustrating our method on selecting neighborhood cells for imputation. However, we acknowledge that all data examined in the present study contain a smaller number of cells than the number of genes. The accuracy of gene-based imputation is likely dependent on the number of cells and will likely improve with increasing cell number. In contrast, the accuracy of cell-based imputation is likely dependent on the number of genes. Subsequently, using genes to perform imputation may have added benefits for larger scRNAseq data where the number of cells exceeds the number of genes. Our method can be easily adapted to switch from cell-based imputation to gene-based imputation for large scRNAseq data. In addition, exploring the benefits of combining both imputation strategies will be an important avenue for future research.

Like all other existing imputation methods [24–27], we have been primarily focused on modeling and imputing log-transformed normalized gene expression data that are converted from the original count data. Modeling log-transformed normalized expression data assumes application of a normalization method as a pre-processing step. Though the performance of normalization methods varies in different settings, VIPER does not depend on choice of a particular normalization method. While in the present study we have only examined a relatively simple normalization method based on RPM, we note that using advanced normalization offsets [40] or including cellular detection rate [41] may further improve the performance of VIPER. Importantly, modeling log-transformed normalized gene expression data using Gaussian models is computationally more tractable than modeling count data using over-dispersed Poisson models (e.g., negative binomial or Poisson mixed models) [42–44]. Because of the computational tractability, modeling log-transformed normalized gene expression data are commonly applied in scRNAseq studies for clustering analysis, differential expression analysis, and various other analytic tasks [20, 22, 45]. However, scRNAseq data are of count nature. Because of the relatively low sequencing depth of scRNAseq, accounting for the mean and variance relationship by modeling the original count data directly often has added benefits [20, 22, 23]. Therefore, extending our method to model and impute the count data from scRNAseq directly while properly accounting for the over-dispersion or dropout events will likely improve imputation accuracy further, especially for data with lower per-cell read depth such as those collected from the 10x genomics platform. In addition, for study designs, whether the bulk tissue sequencing and scRNAseq are applied to same cell content, incorporating bulk data as prior information for imputation of scRNAseq data will likely further improve accuracy. Exploring and benchmarking this strategy will also be a promising direction.

## Additional files

Additional file 1: Details of modeling for the dropout event adjustment and method comparison to scImpute. (PDF 200 kb)

Additional file 2: Additional figures for performance evalution of VIPER. (PDF 13470 kb)

Additional file 3: **Tables S1** and **S2**. Percentage of zero values in the downsampling experiments and computating time comparisons. (PDF 150 kb)
