# Supplementary material for: VIPER: variability-preserving imputation for accurate gene expression recovery in single-cell RNA sequencing studies
Source: Genome Biol. 2018 Nov 12;19:196. doi: 10.1186/s13059-018-1575-1 (PMC6233584; doi:10.1186/s13059-018-1575-1)
Supplement: Supplementary file 1 — Details of modeling for the dropout event adjustment and method comparison to scImpute. (PDF 200 kb) [file 13059_2018_1575_MOESM1_ESM.pdf]

Supplementary Text

1 Modeling and Algorithm Details for the Dropout Event Adjustment

The VIPER imputation procedure involves a step for adjusting dropout events. And we describe the modeling and algorithm details for this dropout event adjustment in details here. We first follow the main notation from the main text to explain the rationales. Specifically, here we aim to distinguish the two possible mechanisms that generate a zero count: a zero count either comes from a dropout event or represents a low or zero level of gene expression. If the zero value of the expression count $C_{l,j}$ is due to a dropout event, then it is not an accurate measurement of the true expression level of j-th gene in l-th cell. Subsequently, we do not wish to use the normalized value $X_{l,j}$ from a dropout event to impute $X_{i,j}$. However, if the zero value of $C_{l,j}$ comes from low or zero expression level of j-th gene in l-th cell, then we would want to use the normalized value $X_{l,j}$ to impute $X_{i,j}$. To distinguish between these two possibilities, we estimate an expected expression level for any zero value of $C_{l,j}$ and use these estimates to perform imputation. In particular, we assume that the gene expression levels of j-th gene for all selected neighborhood cells for the i-th cell of interest follow a zero inflated Poisson mixed model, such that

$$C_{l,j} \sim p_{i,j}\delta_0 + (1 - p_{i,j})PMM(N_l\lambda_{l,j}, \psi_{i,j})$$

where $p_{i,j}$ represents the dropout probability of j-th gene that is specific for all neighborhood cells of the i-th cell of interest; δ_0 denotes a point mass at zero; N_l is the total read depth for the l-th cell; $\lambda_{l,j}$ is the Poisson rate parameter that represents the expression level of j-th gene in the l-th cell; $\psi_{i,j}$ is an over-dispersion parameter that is specific for j-th gene and for all neighborhood cells of the i-th cell of interest; and PMM denotes a Poisson mixed effects model. Under the zero inflated Poisson mixed effects model, $C_{l,j}$ is exactly zero with a dropout probability $p_{i,j}$ and follows an over-dispersed Poisson distribution with probability $1 - p_{i,j}$. Our goal is to estimate $\lambda_{l,j}$, the underlying expression level of j-th gene in l-th cell, to serve as our final predictor variable for all zero values of $C_{l,j}$. To do so, we first estimate all parameters (i.e. $p_{i,j}, \lambda_{l,j}, \psi_{i,j}$) through an expectation maximization (EM) algorithm based on the selected neighborhood cells for the i-th cell of interest.

To describe the EM algorithm in detail, we need to use a different set of notations in order to simplify annotation. Specifically, we ignore the gene subscript and denote

$$Y_l := C_{l,j}, \quad \pi_0 := p_{i,j}, \quad \pi_1 := 1 - \pi_0, \quad \lambda_l := \lambda_{l,j}, \quad \psi := \psi_{i,j}$$

Therefore, the above equation can be re-expressed as

$$Y_l \quad \sim \quad \pi_0 \delta_0 + \pi_1 PMM(\mu, \psi; N_l).$$

where the Poisson mixed model (PMM) is defined as

$$\begin{aligned} &X \sim PMM(\mu, \psi; N_l) \quad \text{is equivalent to} \\ &X \sim \text{Poi}(N_l \lambda_l) \quad \log \lambda_l = \mu + e_l \quad e_l = \log w_l \quad w_l \sim \text{Gamma}(\psi, \psi) \end{aligned}$$

Let I_Y be a vector of latent variables to represent the membership of each data point, which takes value of either 0 or 1. We denote $\mathbf{\Theta} = \{\mu, \psi\}$ and we write down the likelihood as:

$$\begin{aligned} P(Y_l, I_{Y_l} = 1|\mathbf{\Theta}) &= \frac{\psi^\psi}{Y_l!} \frac{\Gamma(Y_l + \psi)}{\Gamma(\psi)} \frac{(N_l e^\mu)^{Y_l}}{(N_l e^\mu + \psi)^{Y_l + \psi}} \\ P(Y_l \neq 0, I_{Y_l} = 0|\mathbf{\Theta}) &= 0 \\ P(Y_l = 0, I_{Y_l} = 0|\mathbf{\Theta}) &= \frac{1}{P(Y_l = 0|I_{Y_l} = 1, \mathbf{\Theta}) + 1} \end{aligned}$$

The marginal likelihood of observing $\mathbf{Y}$ given $\mathbf{I_Y}$ can be written as:

$$\log L(\mathbf{Y}|\mathbf{I_Y}, \mathbf{\Theta}) = \sum_{I_{Y_l}=0} P(Y_l, I_{Y_l} = 0|\mathbf{\Theta}) + \sum_{I_{Y_l}=1} P(Y_l, I_{Y_l} = 1|\mathbf{\Theta})$$

Treating $\mathbf{I_Y}$ as missing data, we develop an EM algorithm to estimate the model parameters. Our expectation step (E-Step) is

$$\begin{aligned} &E_{\mathbf{I_Y}|\mathbf{Y},\Theta} \log L(\mathbf{Y}|\mathbf{I_Y}, \mathbf{\Theta}) = \sum_l \mathbb{P}(I_{Y_l} = 0|\mathbf{Y}, \mathbf{\Theta}) \\ &+ \sum_i \mathbb{P}(I_{Y_l} = 1|\mathbf{Y}, \mathbf{\Theta}) \Big[Y_l \log N_l + Y_l \mu + \psi \log \psi + \log \Gamma(Y_l + \psi) - \log Y_l! - \log \Gamma(\psi) - (Y_l + \psi) \log(N_l e^\mu + \psi) \Big] \end{aligned}$$

We optimize the above quantity by gradient descent in the maximization step (M-Step). To do so, we calculate the gradient at each t-th iteration:

$$\frac{\partial E_{\mathbf{I_Y}|\mathbf{Y},\Theta} \log L(\mathbf{Y}|\mathbf{I_Y}, \mathbf{\Theta})}{\partial \mu} = \sum_i \mathbb{P}(I_{Yi} = 1|\mathbf{Y}, \mathbf{\Theta}) \Big[Y_i - (Y_i + \psi) \frac{N_l e^\mu}{N_l e^\mu + \psi} \Big]$$

$$\frac{\partial E_{\mathbf{I_Y}|\mathbf{Y},\Theta} \log L(\mathbf{Y}|\mathbf{I_Y}, \mathbf{\Theta})}{\partial \psi}$$

$$= \sum_i \mathbb{P}(I_{Yi} = 1|\mathbf{Y}, \mathbf{\Theta}) \Big[\log \psi + 1 - \frac{Y_i + \psi}{N_l e^\mu + \psi} - \log(N_l e^\mu + \psi) + \text{digamma}(Y_i + \psi) - \text{digamma}(\psi) \Big]$$

$$\pi_k^{(t+1)} = \frac{\sum_i P(I_{Y_l} = k|\mathbf{Y}, \mathbf{\Theta}^{(t+1)})}{\#\{\mathbf{Y}\}}$$

where $k = 0$ or 1. With these derivatives, we update all parameters using a gradient descent algorithm. Afterwards, at E-Step, we update the posterior likelihood as:

$$\mathbb{P}(I_{Y_l} = k|\mathbf{Y}, \mathbf{\Theta}^{(t+1)}) = \frac{\pi_k^{(t)} P(Y_l|I_{Y_l} = k, \mathbf{\Theta}^{(t+1)})}{\sum_k \pi_k^{(t)} P(Y_l|I_{Y_l} = k, \mathbf{\Theta}^{(t+1)})}$$

We iterate through the E-Step and M-Step until convergence. Afterwards, we obtain an estimate for λ_l (i.e. $\lambda_{l,j}$) based on these parameter estimates. We use the posterior mean estimate $\hat{\lambda}_{l,j}$ to replace zero $C_{l,j}$ to serve as the final predictor variable. Certainly, we use the normalized expression measurement $X_{l,j}$ directly as the predictor variable if $C_{l,j}$ is non-zero.

2 Differences between VIPER and scImpute

There are three key differences between VIPER and scImpute:

(1) Model and parameter specification. scImpute requires knowing a priori the number of cell subpopulations (k) in the data, which is unfortunately not known in any real data. Importantly, as we have shown in the Discussion section and the Supplementary Figures S24-S25, the performance of scImpute is sensitive to the pre-specified number of cell subpopulations and depends heavily on k. For example, the clustering results for the H1 cell subpopulation from the Cell Type data using the scImpute imputed data are sensitive to k. In particular, if we set the number of cell subpopulations in the H1 cell type to be either 1, 2, $\cdots$ or 6 before imputation, then we would also detect an increasing number of (artificial) cell subpopulations after imputation. In contrast, our method does not require the pre-specification of the true number of cell subpopulations or any other tuning parameters. Instead, we infer all parameters from the data at hand. Subsequently, our method yields much more stable and accurate results compared with scImpute for various data sets. For example, the clustering results for the H1 cell subpopulation using the VIPER imputed data are generally consistent with that using the raw data, suggesting a single homogeneous cell subpopulation in H1 cell type, as one might expect.

(2) Selection of predictive cells. scImpute only selects neighborhood cells from the same subpopulation to predict the cell of interest. Subsequently, as explained in (1), the results of scImpute are sensitive to how the subpopulation were inferred. In addition, because the cells of interest in the same cell subpopulation are predicted using the same, and likely small, subset of cells within the same population, the predicted expression levels across cells

within the same population may suffer from low expression variability. Indeed, from the CV analysis results (Figure 5C and Supplementary Figures S8-S13), we have confirmed that the expression variability across cells was reduced considerably in scImpute imputed data as expected. In contrast, our method selects the predictive cells from all cells and is thus much less likely to suffer from the same shortcoming of scImpute.

(3) Treatment of drop-out events. scImpute estimates the drop-out probability for one gene at a time using all cells within a given cell subpopulation. Depending on this estimated probability, scImpute will determine whether or not to impute this particular gene for all cells within the cell subpopulation. Therefore, the performance of scImpute will depend heavily on the estimated dropout probability and the cut-off: if the dropout probability is not estimated accurately, then scImpute will either incorrectly impute all cells within the subpopulation or incorrectly impute none of them, resulting in suboptimal performance. In contrast, we follow the main idea of SAVER and impute all zero values regardless whether it is a drop-out event or not. We reason that, if a zero value in a given gene and cell is due to low abundance and true zero expression levels, then the predictive cells for this cell of interest will also contain a large number of low or zero expression values. Subsequently, the resulting prediction for the true zero or low expression value will remain small. If a zero value in a given gene and cell is due to dropout, then the predictive cells for this cell of interest will tend to contain a large number of non-zero or large expression values. Subsequently, the resulting prediction value will remain large. In addition, we estimate the drop-out probabilities for all neighborhood cells and use these probabilities to adjust for these explanatory/predictive variables, which further improves imputation accuracy. We model the drop-out probability for each cell of interest separately using only its neighborhood cells to ensure that such adjustment is carried out in a cell specific fashion. Indeed, as shown in the real-data based experiments (e.g. Figure 3 and Supplementary Figure S6), our approach does yield more accurate imputation results than scImpute even for zero values that are truly due to low abundance.
