# Supplementary material for: VIPER: variability-preserving imputation for accurate gene expression recovery in single-cell RNA sequencing studies
Source: Genome Biol. 2018 Nov 12;19:196. doi: 10.1186/s13059-018-1575-1 (PMC6233584; doi:10.1186/s13059-018-1575-1)
Supplement: Supplementary file 2 — Additional figures for performance evalution of VIPER. (PDF 13470 kb) [file 13059_2018_1575_MOESM2_ESM.pdf]

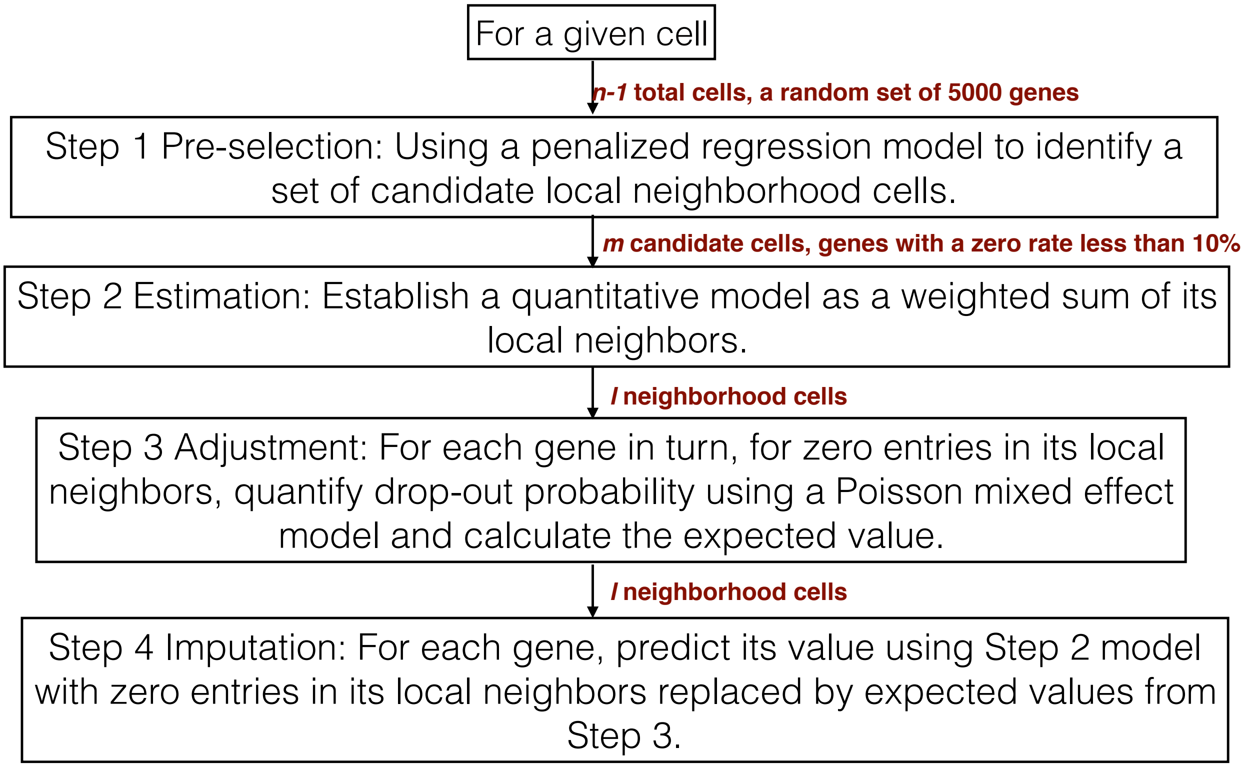

Fig S1. Illustration of the VIPER imputation procedure. VIPER relies on two main steps -- pre-selection and estimation – to progressively select a small set of neighborhood cells for imputing the cell of interest. After the selection of neighborhood cells, VIPER further accounts for dropout events in the neighborhood cells with an additional predictor variable adjustment step. Afterwards, VIPER imputes zeros in the cell of interest with the predator variables and parameters obtained from the first three steps.

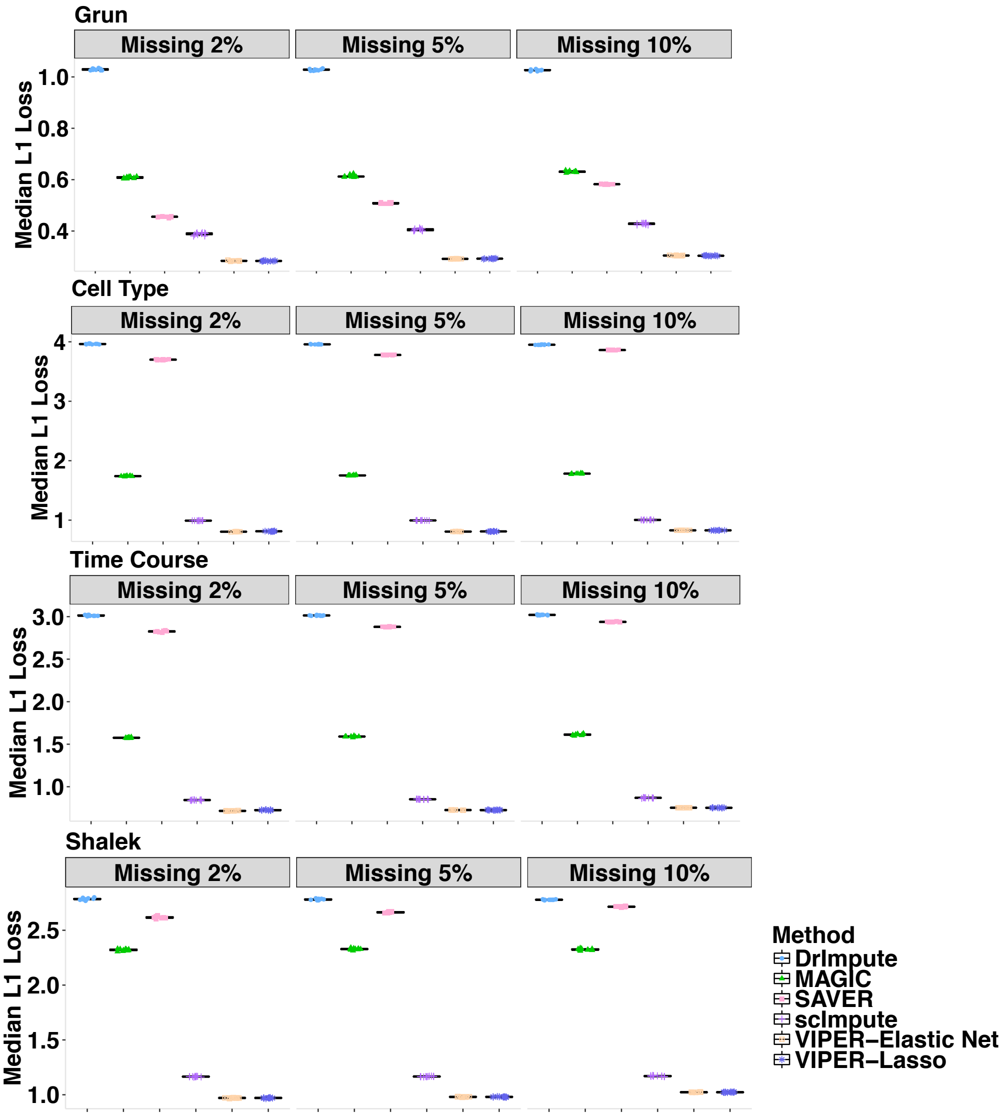

Fig S2-S4. Accuracy of the imputed values by different methods in the data masking experiment. Accuracy is measured by median L1 loss (**S2**), mean square loss (**S3**), or mean L1 loss (**S4**) as compared to the masked truth. Rows represent the four different data sets (Grun, Cell Type, Time Course and Shalek) used in the experiment. Columns represent masking percentage (2%, 5%, and 10%). Methods for comparison include DrImpute (blue), MAGIC (green), SAVER (pink), scImpute (purple), VIPER with elastic net selection (peach), and VIPER with lasso selection (dark blue). Boxplots show values obtained from 10 masking replicates, where in each replicate we calculated an measure of imputation accuracy for each cell in turn and plotted the median value across cells.

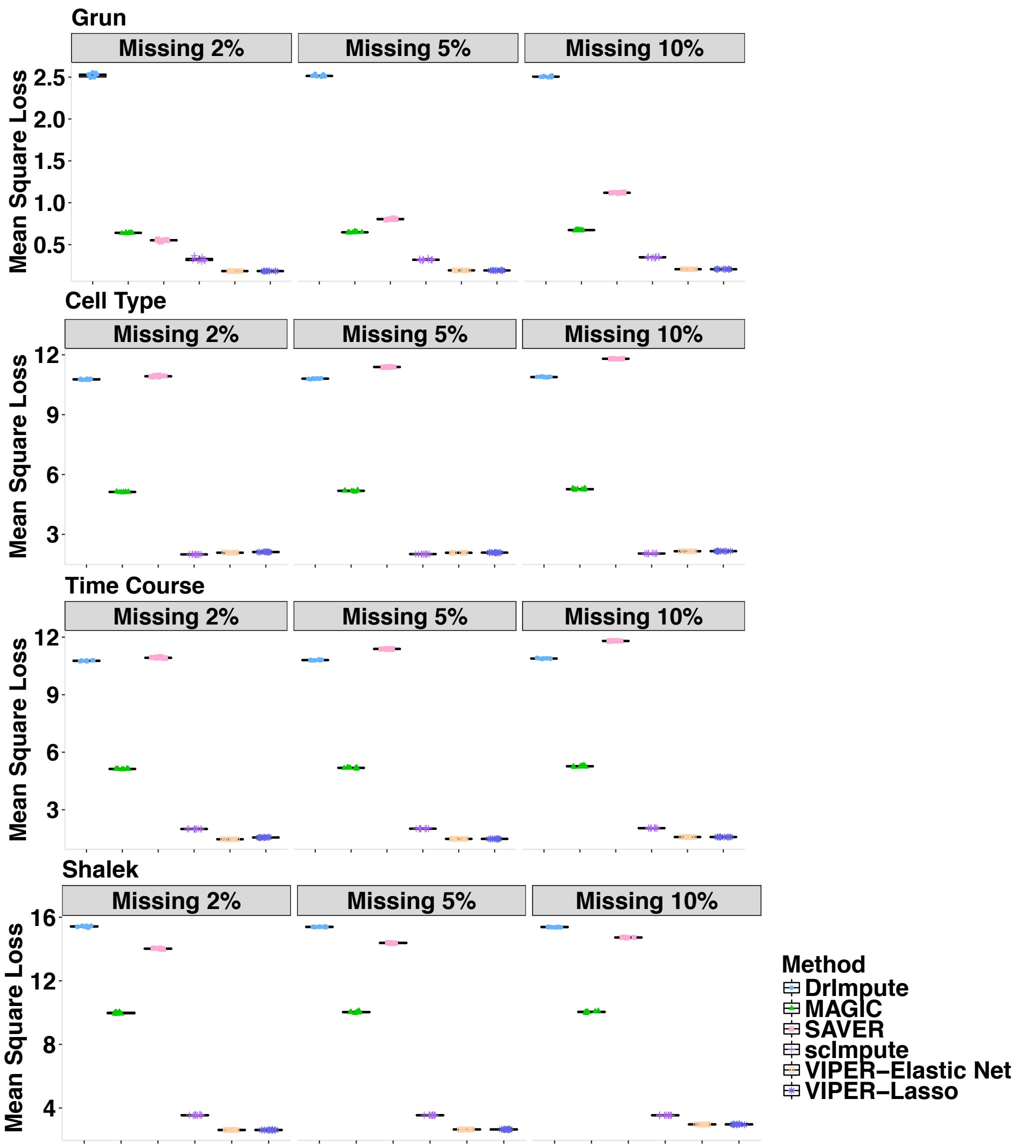

Fig S2-S4. Accuracy of the imputed values by different methods in the data masking experiment. Accuracy is measured by median L1 loss (**S2**), mean square loss (**S3**), or mean L1 loss (**S4**) as compared to the masked truth. Rows represent the four different data sets (Grun, Cell Type, Time Course and Shalek) used in the experiment. Columns represent masking percentage (2%, 5%, and 10%). Methods for comparison include DrImpute (blue), MAGIC (green), SAVER (pink), scImpute (purple), VIPER with elastic net selection (peach), and VIPER with lasso selection (dark blue). Boxplots show values obtained from 10 masking replicates, where in each replicate we calculated an measure of imputation accuracy for each cell in turn and plotted the median value across cells.

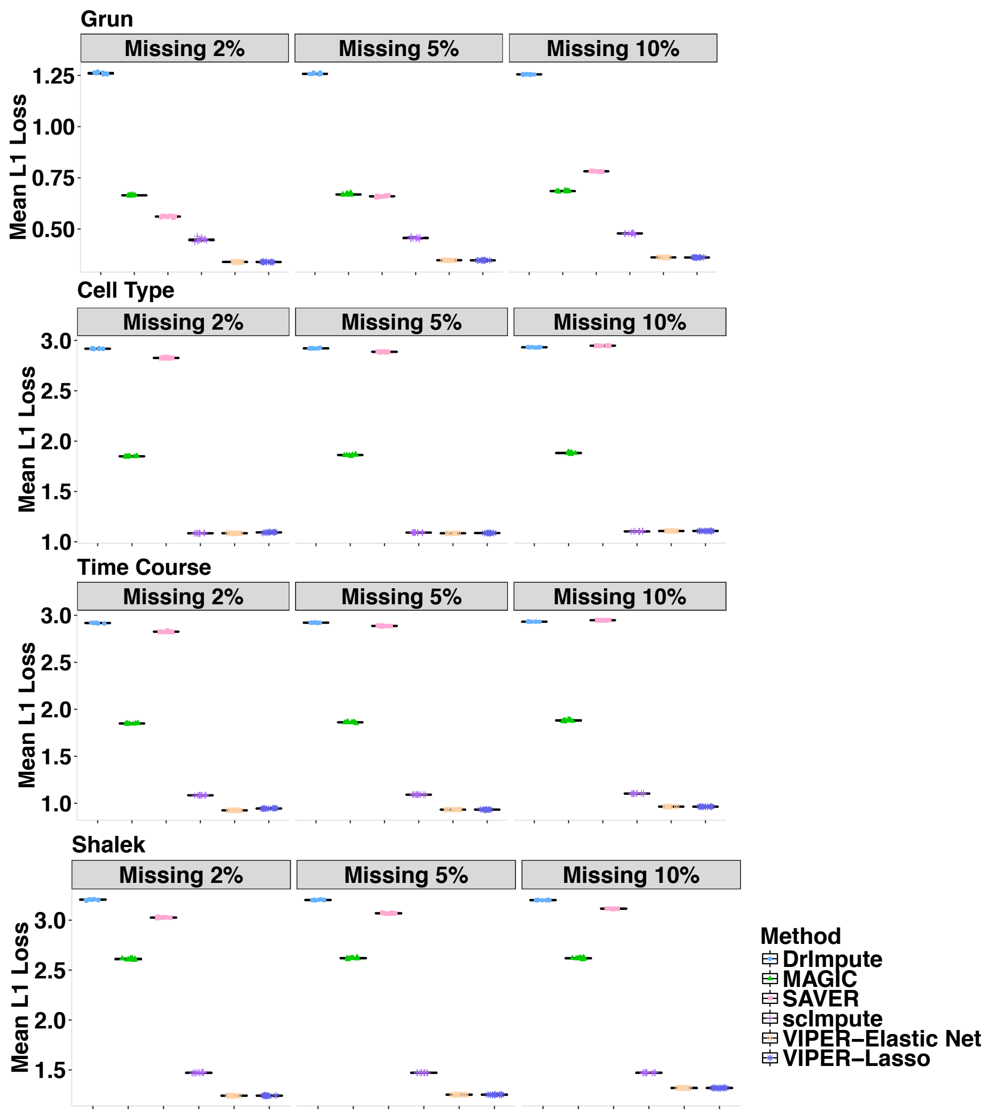

Fig S2-S4. Accuracy of the imputed values by different methods in the data masking experiment. Accuracy is measured by median L1 loss (**S2**), mean square loss (**S3**), or mean L1 loss (**S4**) as compared to the masked truth. Rows represent the four different data sets (Grun, Cell Type, Time Course and Shalek) used in the experiment. Columns represent masking percentage (2%, 5%, and 10%). Methods for comparison include DrImpute (blue), MAGIC (green), SAVER (pink), scImpute (purple), VIPER with elastic net selection (peach), and VIPER with lasso selection (dark blue). Boxplots show values obtained from 10 masking replicates, where in each replicate we calculated an measure of imputation accuracy for each cell in turn and plotted the median value across cells.

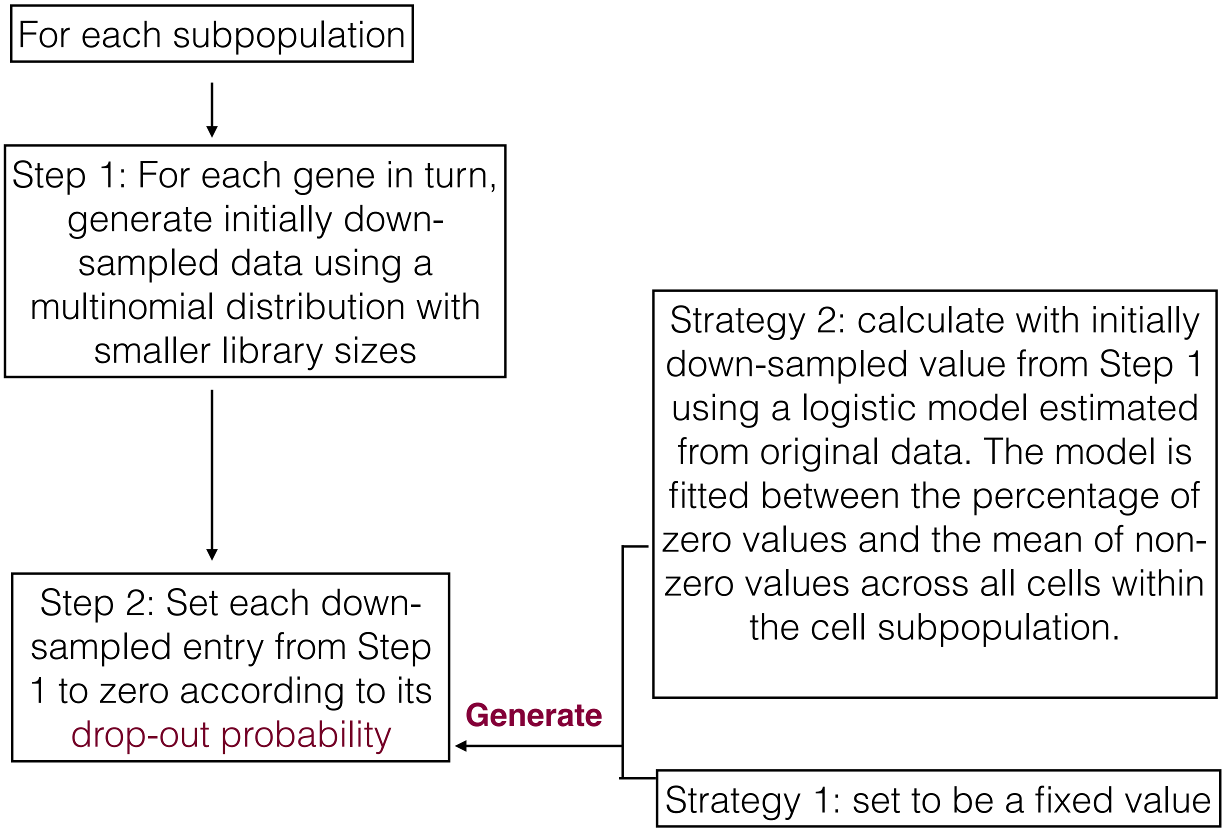

Fig S5. Illustration of the down-sampling experiment. The down-sampling experiment involves two main steps: a multinomial down-sampling step and an extra dropout step. The dropout event is introduced on none-zero values either using a fixed dropout rate that is not dependent on the expression level (strategy 1) or using a dropout rate dependent on the expression level (strategy 2).

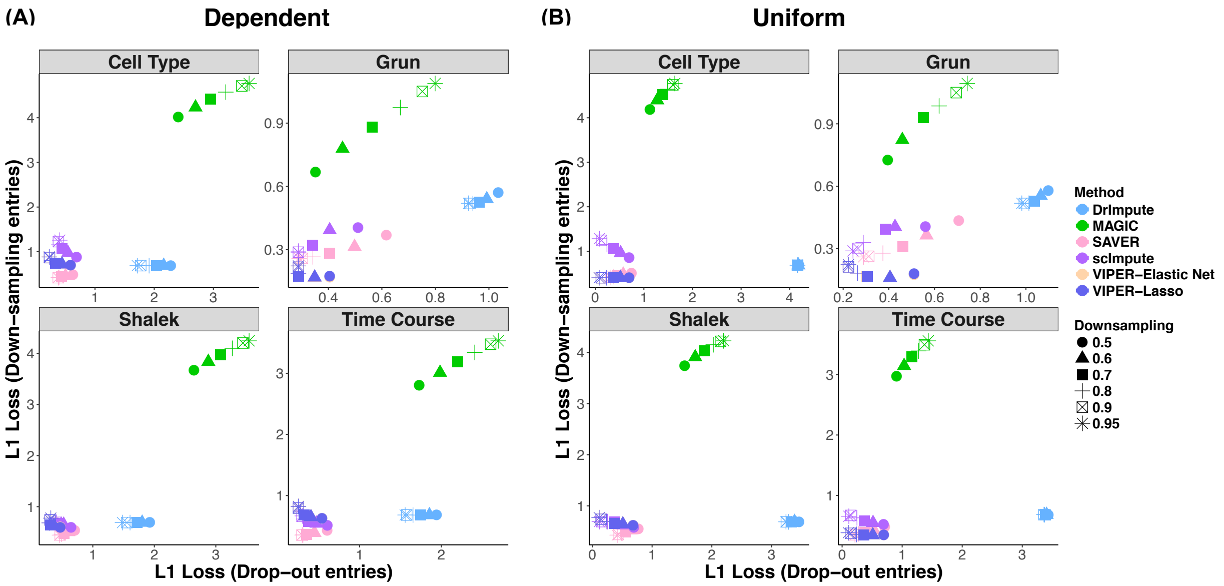

Fig S6: Imputation accuracy in the down-sampling experiment. Results are shown for down-sampling experiments using either expression dependent dropout rate (**A**) or expression independent dropout rate (**B**) for four different datasets (Gurn, Cell Type, Time Course, and Shalek). Imputation accuracy are measured by comparing imputed values to the original truth and are evaluated for two different types of zeros separately: zeros that are due to low expression level in the original data and the multinomial subsampling step (down-sampling entries; y-axis), and zeros that are due to dropout events (dropout entries; x-axis). Accuracy is measured by L1 loss for the dropout entries and by L1 loss for the down-sampling entries. Color of the dots represent methods for comparison that include DrImpute (blue), MAGIC (green), SAVER (pink), scImpute (purple), VIPER with elastic net selection (peach), and VIPER with lasso selection (dark blue). Shape of the dots represent the down-sampling rate used in the multinomial subsampling step.

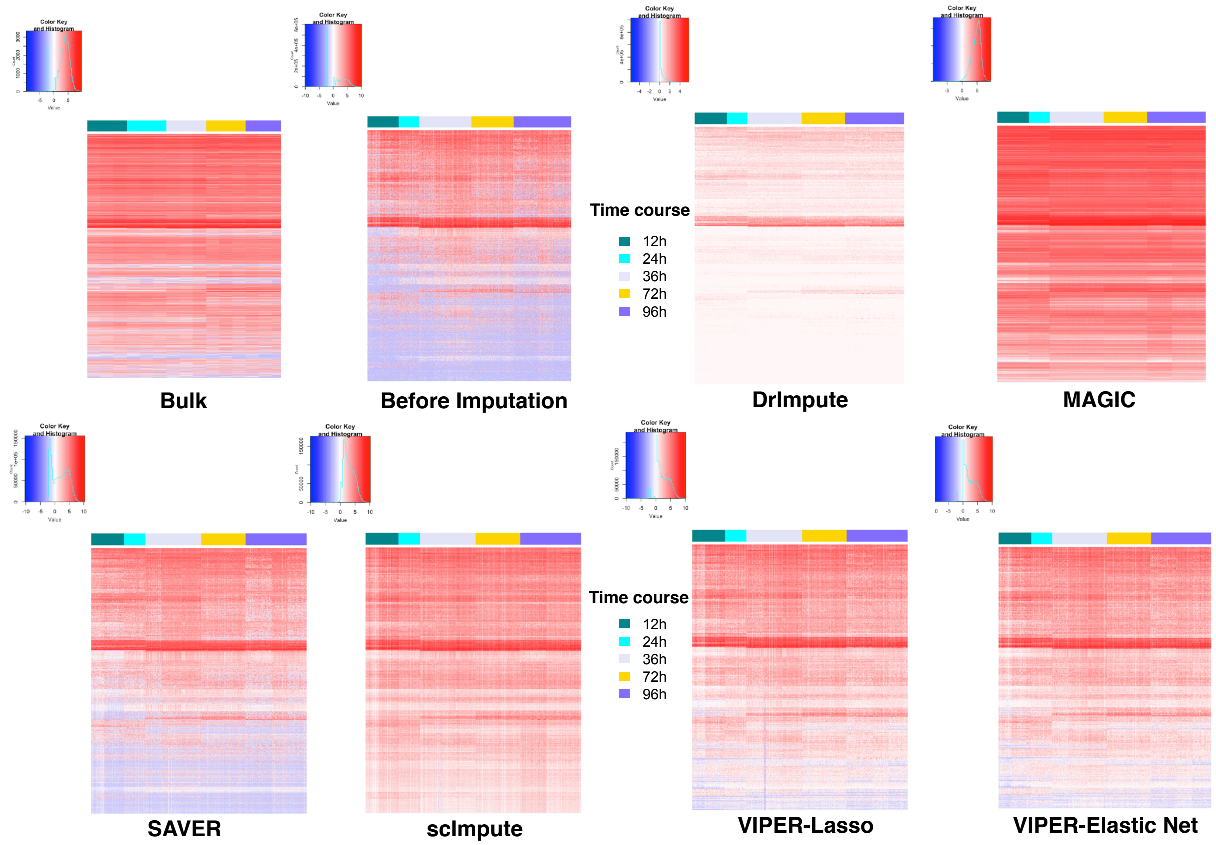

Fig S7: Heatmaps show the unimputed or imputed gene expression measurements in the scRNAseq data together with the gene expression measurements from bulk RNAseq in the Time Course data. Expression measurements are shown across cells (for scRNAseq) or across sample replicates (for bulk RNAseq) in five different time points. The five different time points include 12h, 24h, 36h, 72h, and 96h. Imputed scRNAseq data are obtained from different imputation methods that include DrImpute, MAGIC, SAVER, scImpute, VIPER with elastic net selection, and VIPER with lasso selection.

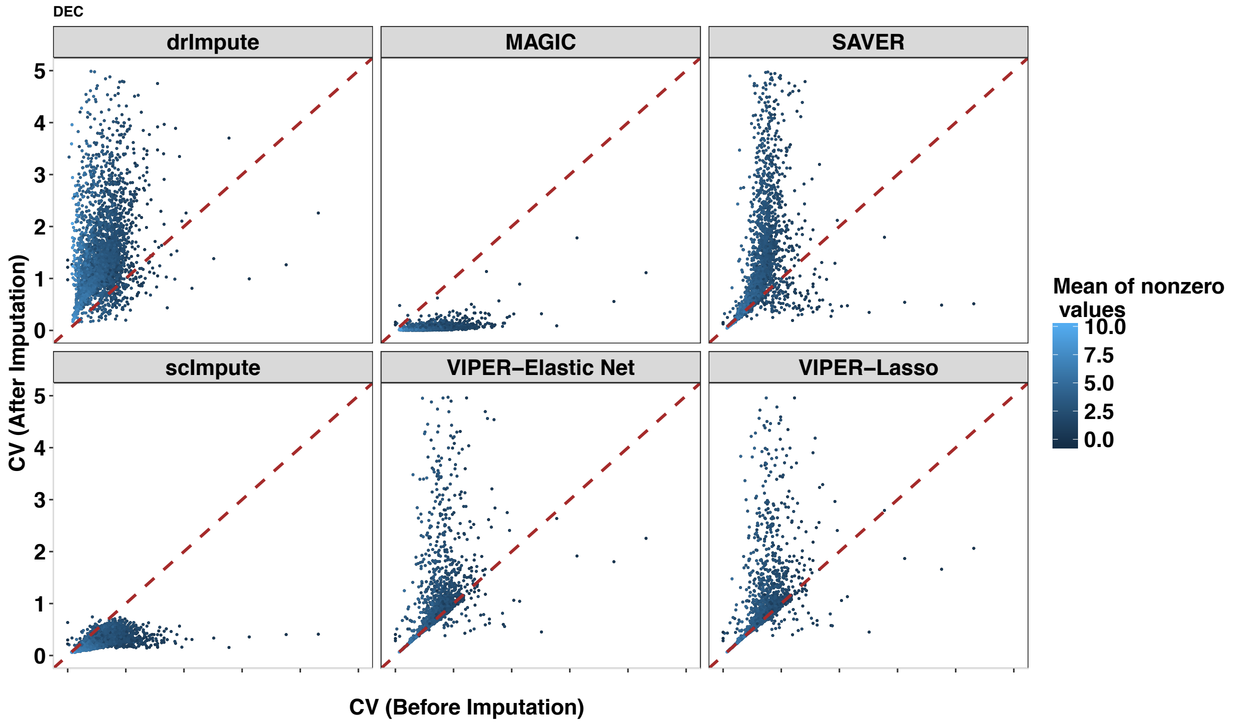

Fig S8-S11: Comparison of gene expression variation across cells in imputed data versus unimputed data. Results are show for DEC cells in Cell Type data (**S8**), stem cells in the 2i culture medium in the Grun data (**S9**), H9 cell after 24hr in the Time Course data (**S10**), and 6hr LPS treated cells in the Shalek data (**S11**). The coefficient of variation (CV) across all cells after imputation (y-axis) is plotted against the CV of non-zero cells before imputation (x-axis) for different methods. Each dot represents a gene and the color of the dot represent the mean of non-zero values. Methods for comparison include DrImpute, MAGIC, SAVER, scImpute, VIPER with elastic net selection, and VIPER with lasso selection. Different non-zero mean expression levels are shown by colors in gradient.

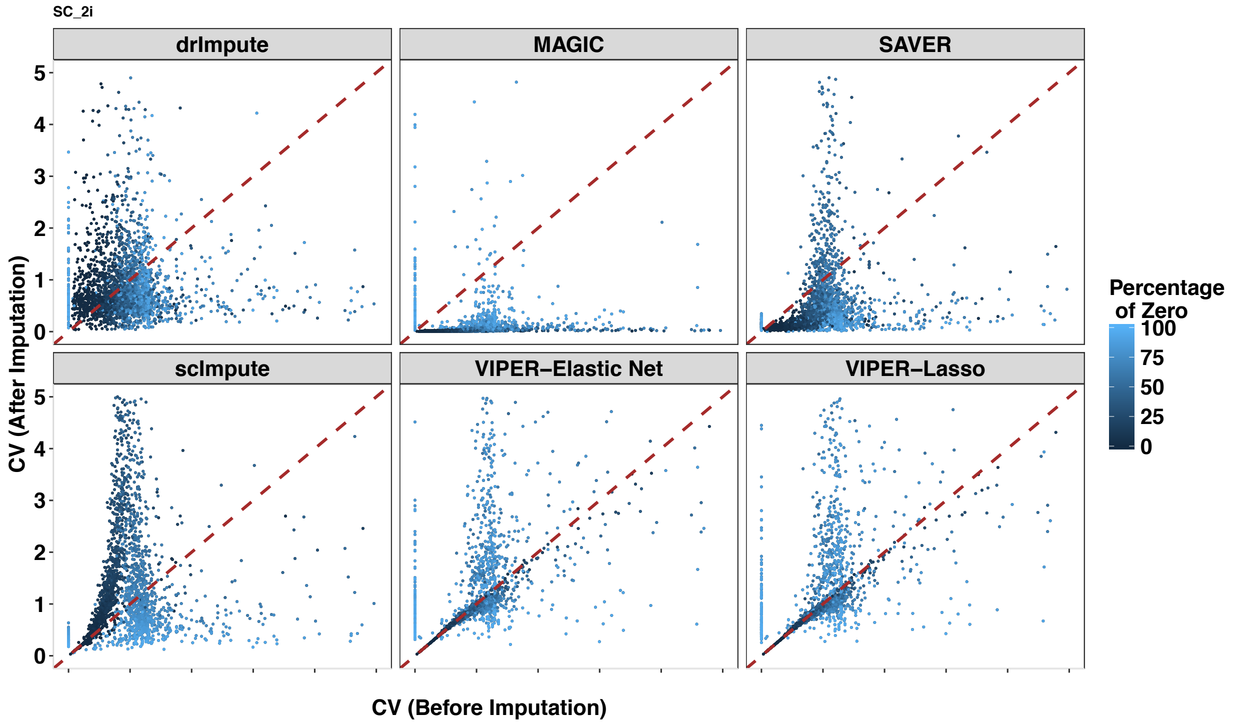

Fig S8-S11: Comparison of gene expression variation across cells in imputed data versus unimputed data. Results are show for DEC cells in Cell Type data (**S8**), stem cells in the 2i culture medium in the Grun data (**S9**), H9 cell after 24hr in the Time Course data (**S10**), and 6hr LPS treated cells in the Shalek data (**S11**). The coefficient of variation (CV) across all cells after imputation (y-axis) is plotted against the CV of non-zero cells before imputation (x-axis) for different methods. Each dot represents a gene and the color of the dot represent the mean of non-zero values. Methods for comparison include DrImpute, MAGIC, SAVER, scImpute, VIPER with elastic net selection, and VIPER with lasso selection. Different non-zero mean expression levels are shown by colors in gradient.

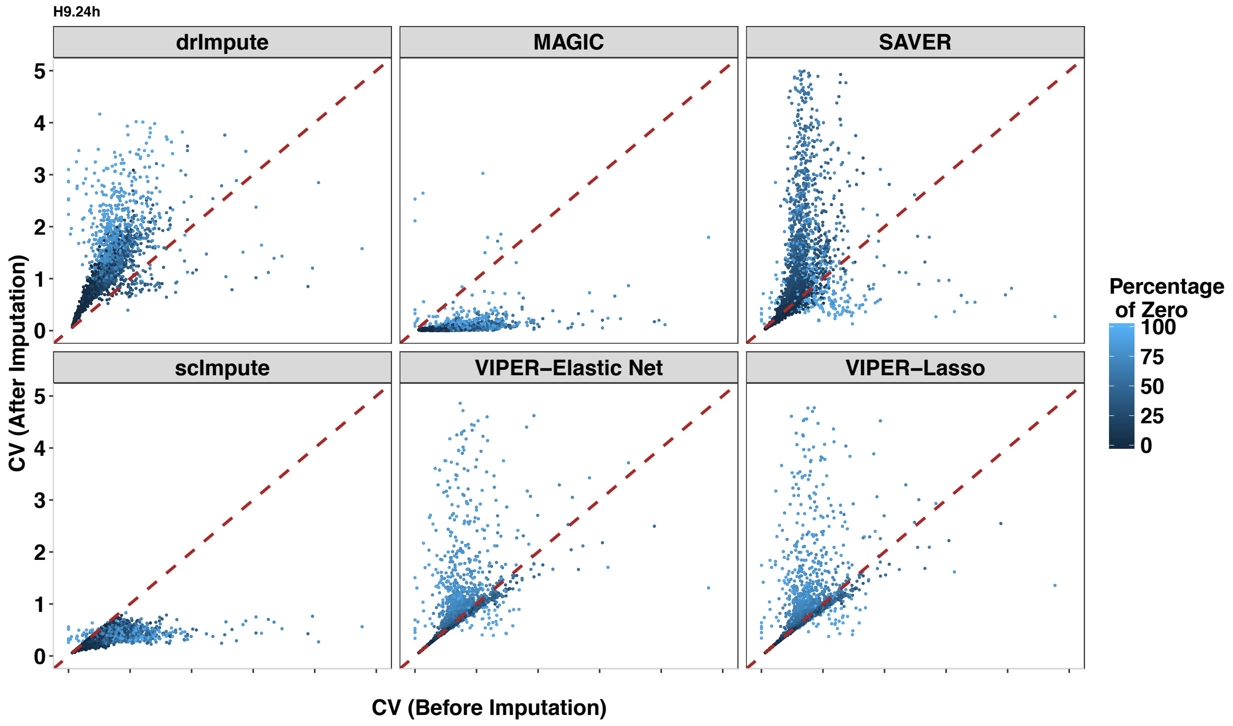

Fig S8-S11: Comparison of gene expression variation across cells in imputed data versus unimputed data. Results are show for DEC cells in Cell Type data (**S8**), stem cells in the 2i culture medium in the Grun data (**S9**), H9 cell after 24hr in the Time Course data (**S10**), and 6hr LPS treated cells in the Shalek data (**S11**). The coefficient of variation (CV) across all cells after imputation (y-axis) is plotted against the CV of non-zero cells before imputation (x-axis) for different methods. Each dot represents a gene and the color of the dot represent the mean of non-zero values. Methods for comparison include DrImpute, MAGIC, SAVER, scImpute, VIPER with elastic net selection, and VIPER with lasso selection. Different non-zero mean expression levels are shown by colors in gradient.

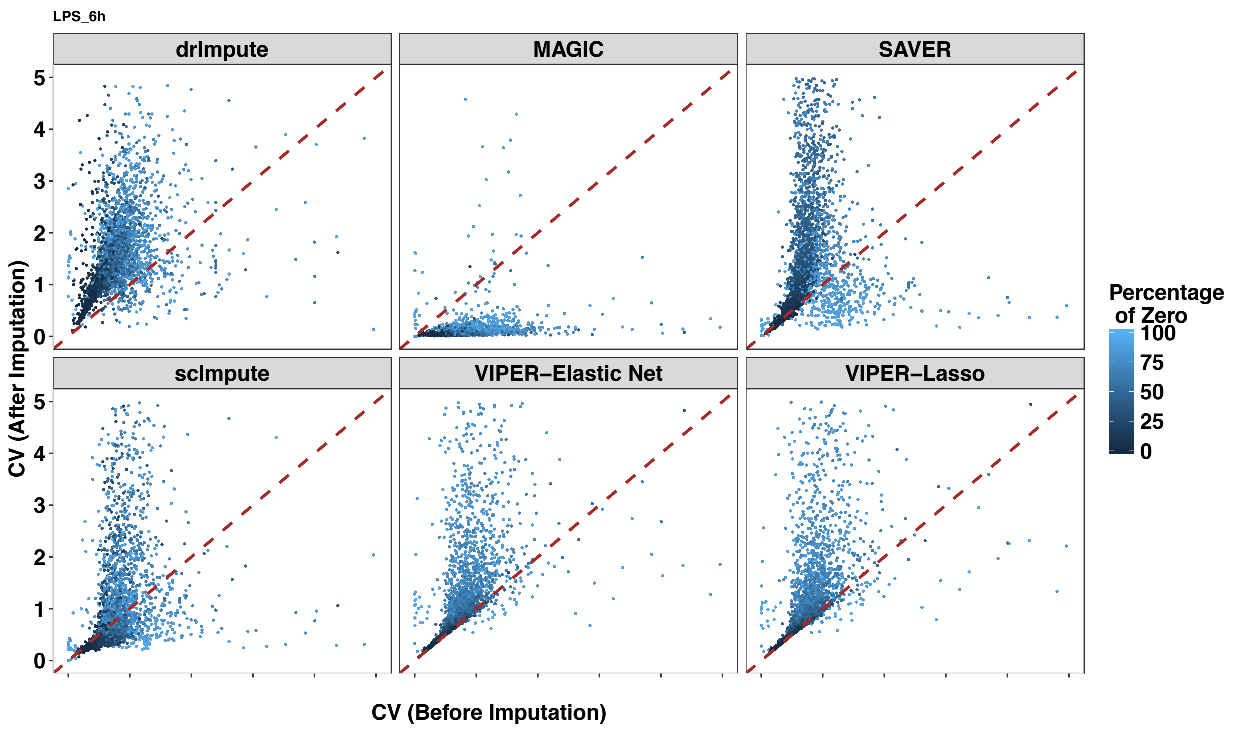

Supplementary Figure S8-S11: Comparison of gene expression variation across cells in imputed data versus unimputed data. Results are show for DEC cells in Cell Type data (**S8**), stem cells in the 2i culture medium in the Grun data (**S9**), H9 cell after 24hr in the Time Course data (**S10**), and 6hr LPS treated cells in the Shalek data (**S11**). The coefficient of variation (CV) across all cells after imputation (y-axis) is plotted against the CV of non-zero cells before imputation (x-axis) for different methods. Each dot represents a gene and the color of the dot represent the mean of non-zero values. Methods for comparison include DrImpute, MAGIC, SAVER, scImpute, VIPER with elastic net selection, and VIPER with lasso selection. Different non-zero mean expression levels are shown by colors in gradient.

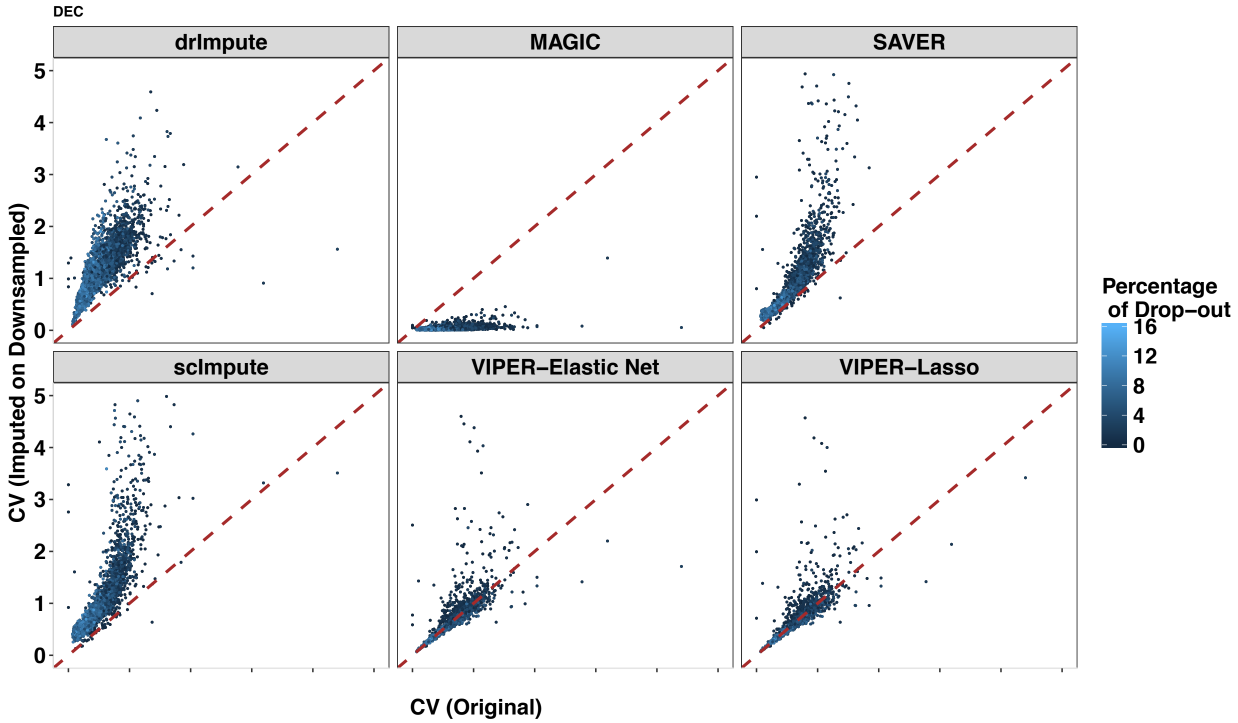

Fig S12-S13: Comparison of gene expression variation across cells in imputed data versus unimputed data in the down-sampling experiments. Results are based on the DEC cells in the Cell Type data using the uniform dropout strategy with a down-sampling rate of 0.8 and are shown for two different types of zeros separately. Specifically, in **S12**, the coefficient of variation (CV) across unimputed values and imputed values for the zeros due to dropout events (y-axis) is plotted against the CV of the corresponding original values before imputation (x-axis) for different methods. In **S13**, the coefficient of variation (CV) across unimputed values and imputed values for the zeros due to low expression levels and subsequent multinomial sampling (y-axis) is plotted against the CV of the corresponding original values before imputation (x-axis) for different methods. Each dot represents a gene and the color of the dot represents either the percentage of dropout zeros (**S12**) or the percentage of down-sampling zeros (**S13**). Methods for comparison include DrImpute, MAGIC, SAVER, scImpute, VIPER with elastic net selection, and VIPER with lasso selection.

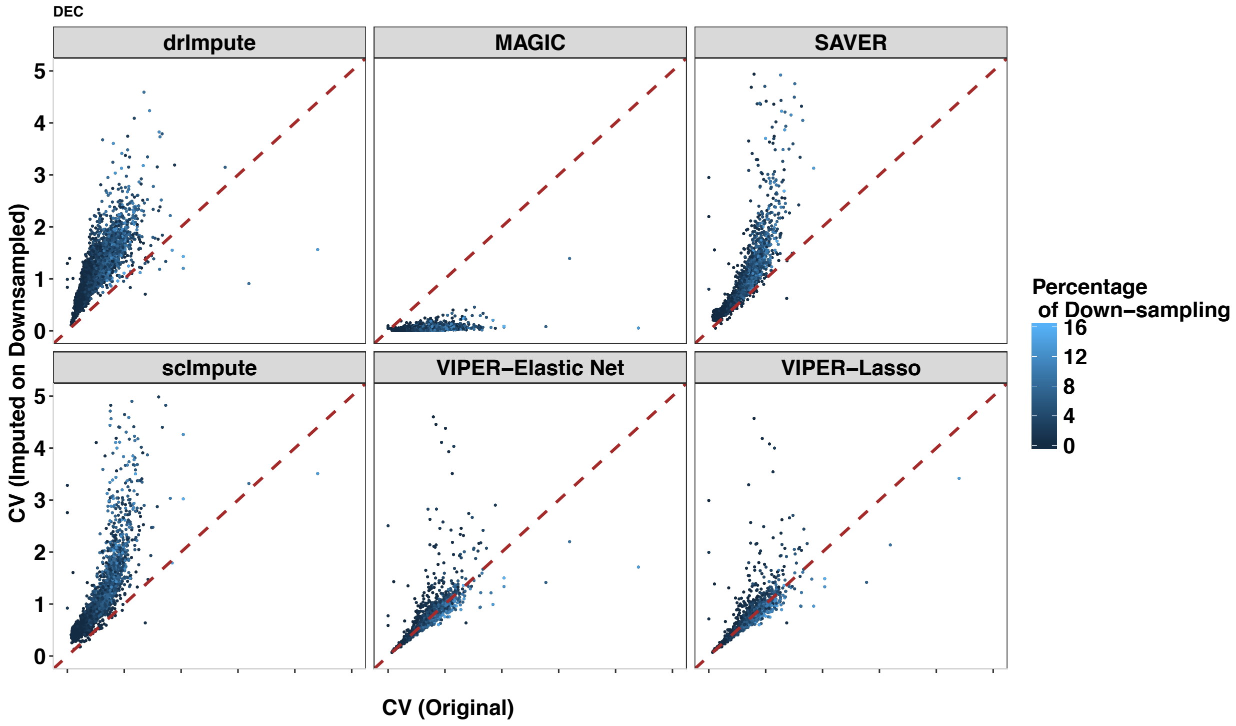

Fig S12-S13: Comparison of gene expression variation across cells in imputed data versus unimputed data in the down-sampling experiments. Results are based on the DEC cells in the Cell Type data using the uniform dropout strategy with a down-sampling rate of 0.8 and are shown for two different types of zeros separately. Specifically, in **S12**, the coefficient of variation (CV) across unimputed values and imputed values for the zeros due to dropout events (y-axis) is plotted against the CV of the corresponding original values before imputation (x-axis) for different methods. In **S13**, the coefficient of variation (CV) across unimputed values and imputed values for the zeros due to low expression levels and subsequent multinomial sampling (y-axis) is plotted against the CV of the corresponding original values before imputation (x-axis) for different methods. Each dot represents a gene and the color of the dot represents either the percentage of dropout zeros (**S12**) or the percentage of down-sampling zeros (**S13**). Methods for comparison include DrImpute, MAGIC, SAVER, scImpute, VIPER with elastic net selection, and VIPER with lasso selection.

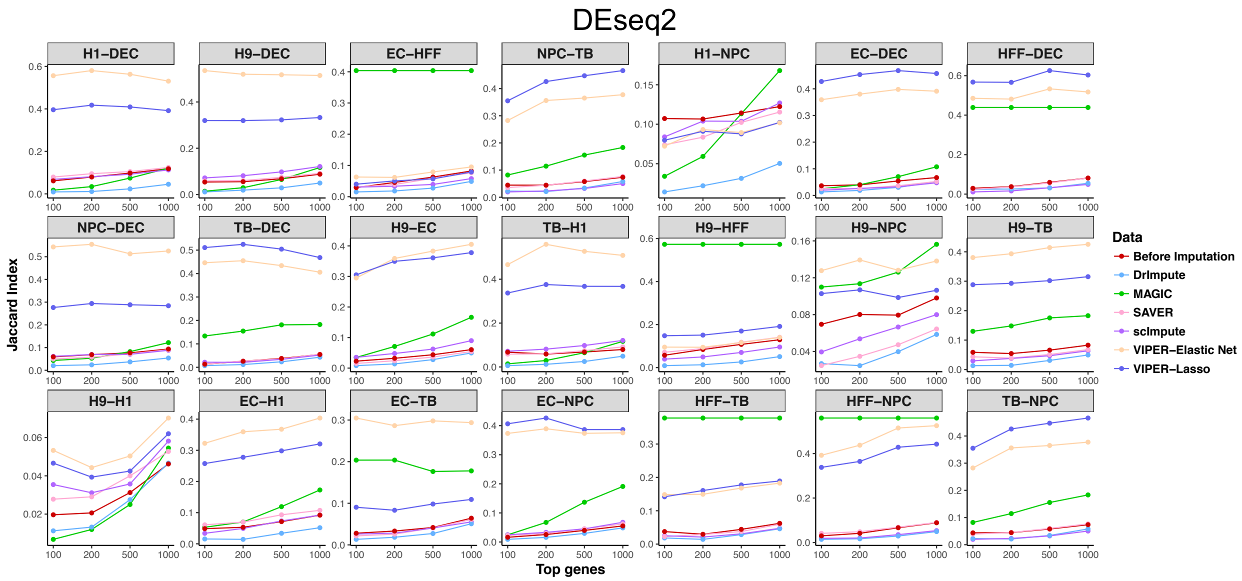

Fig S14-16: Overlap of top differentially expressed genes identified by DEseq2 (**S14**), edgeR-LRT (**S15**) or edgeR-QLF (**S16**) between two data splits, respectively. Each DE method is applied to detect genes that are differentially expressed between pairs of cell subpopulations in the Cell Type data for all pairs of seven cell types. In each comparison, cells from the two cell types are split randomly into two subsets. Imputation and differential expression analysis methods are applied to each data subset separately. The mean Jaccard index between the top 100, 200, 500 or 1000 differentially expressed genes from two subsets are computed across 10 random data splits for each imputation method as a quantification of imputation accuracy, where the Jaccard index is computed as the ratio of the intersection and the union between the top gene lists from the two subsets. Methods for comparison include Before Imputation(red), DrImpute (blue), MAGIC (green), SAVER (pink), scImpute (purple), VIPER with elastic net selection (peach), and VIPER with lasso selection (dark blue).

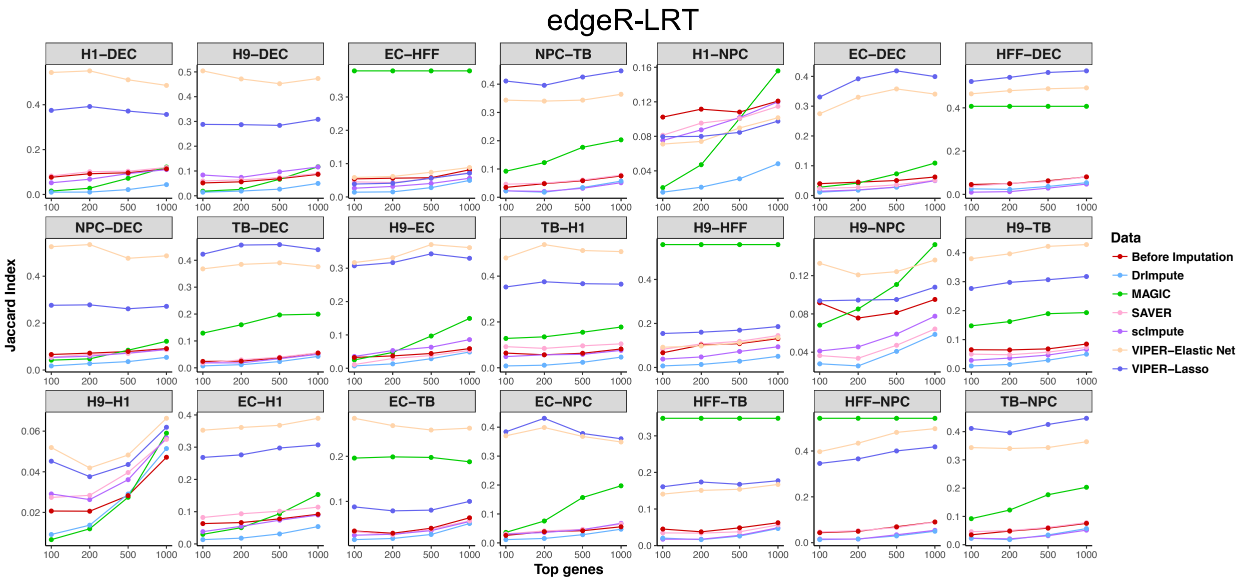

Fig S14-16: Overlap of top differentially expressed genes identified by DEseq2 (**S14**), edgeR-LRT (**S15**) or edgeR-QLF (**S16**) between two data splits, respectively. Each DE method is applied to detect genes that are differentially expressed between pairs of cell subpopulations in the Cell Type data for all pairs of seven cell types. In each comparison, cells from the two cell types are split randomly into two subsets. Imputation and differential expression analysis methods are applied to each data subset separately. The mean Jaccard index between the top 100, 200, 500 or 1000 differentially expressed genes from two subsets are computed across 10 random data splits for each imputation method as a quantification of imputation accuracy, where the Jaccard index is computed as the ratio of the intersection and the union between the top gene lists from the two subsets. Methods for comparison include Before Imputation(red), DrImpute (blue), MAGIC (green), SAVER (pink), scImpute (purple), VIPER with elastic net selection (peach), and VIPER with lasso selection (dark blue).

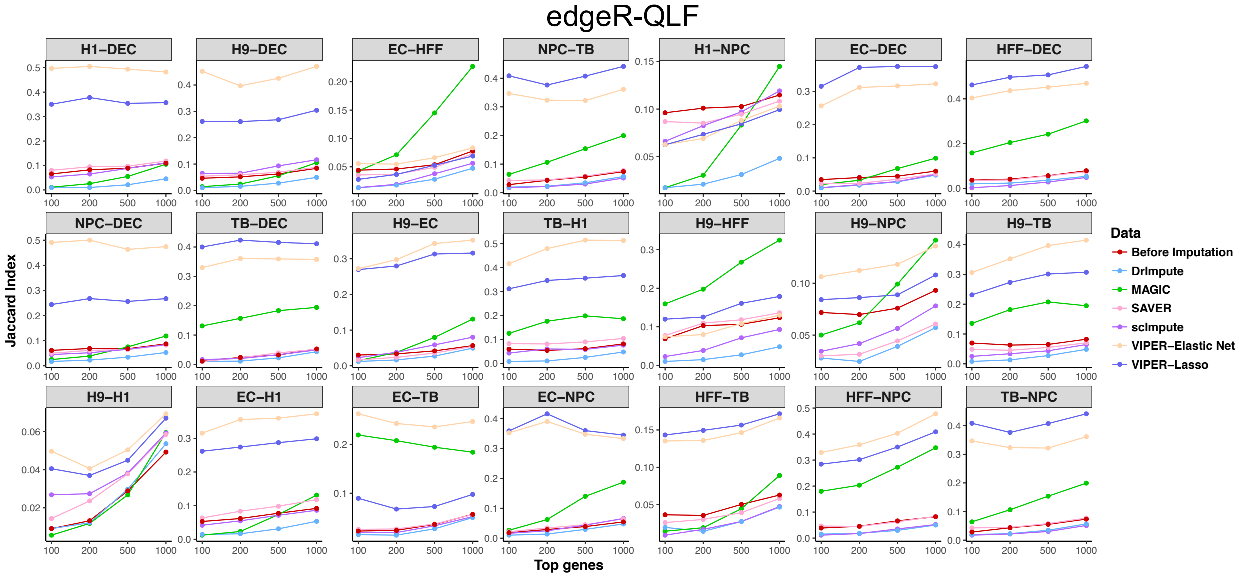

Fig S14-16: Overlap of top differentially expressed genes identified by DEseq2 (**S14**), edgeR-LRT (**S15**) or edgeR-QLF (**S16**) between two data splits, respectively. Each DE method is applied to detect genes that are differentially expressed between pairs of cell subpopulations in the Cell Type data for all pairs of seven cell types. In each comparison, cells from the two cell types are split randomly into two subsets. Imputation and differential expression analysis methods are applied to each data subset separately. The mean Jaccard index between the top 100, 200, 500 or 1000 differentially expressed genes from two subsets are computed across 10 random data splits for each imputation method as a quantification of imputation accuracy, where the Jaccard index is computed as the ratio of the intersection and the union between the top gene lists from the two subsets. Methods for comparison include Before Imputation(red), DrImpute (blue), MAGIC (green), SAVER (pink), scImpute (purple), VIPER with elastic net selection (peach), and VIPER with lasso selection (dark blue).

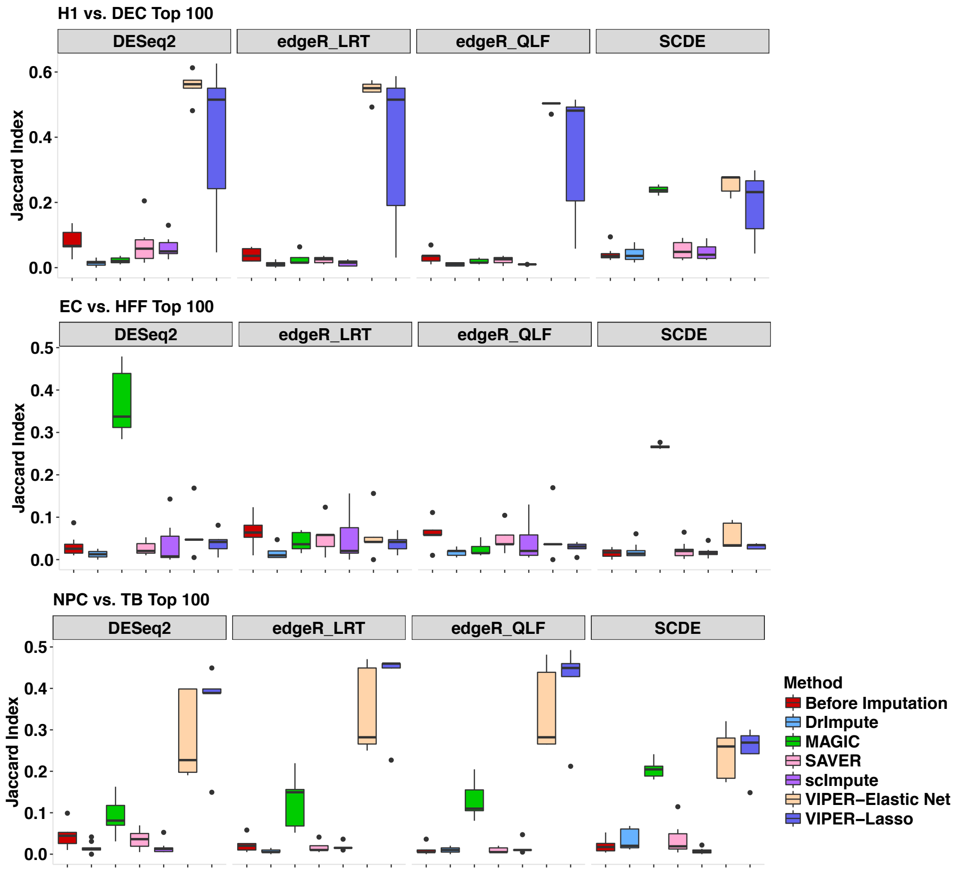

Fig S17: VIPER imputation facilitates the robust detection of differentially expressed genes. Differentially expression tools that include DESeq2 (first column), edgeR-LRT (second column), edgeR-QLF (third column) or SCDE (fourth column) are applied to detect genes that are differentially expressed between two cell subpopulations in the Cell Type data. The compared cell subpopulations include H1 vs DEC (first row), EC vs HFF (second row), or NPC vs TB (third row). In each comparison, the expression data are split randomly into two subsets. Imputation and differential expression analysis methods are applied to each data subset separately. The Jaccard index between the top 100 differentially expressed genes from two subsets are computed across 10 random data splits for each imputation method as a quantification of imputation accuracy, where the Jaccard index is computed as the ratio of the intersection and the union between the top gene lists from the two subsets. Imputation methods for comparison include DrImpute (blue), MAGIC (green), SAVER (pink), scImpute (purple), VIPER with elastic net selection (peach), and VIPER with lasso selection (dark blue).

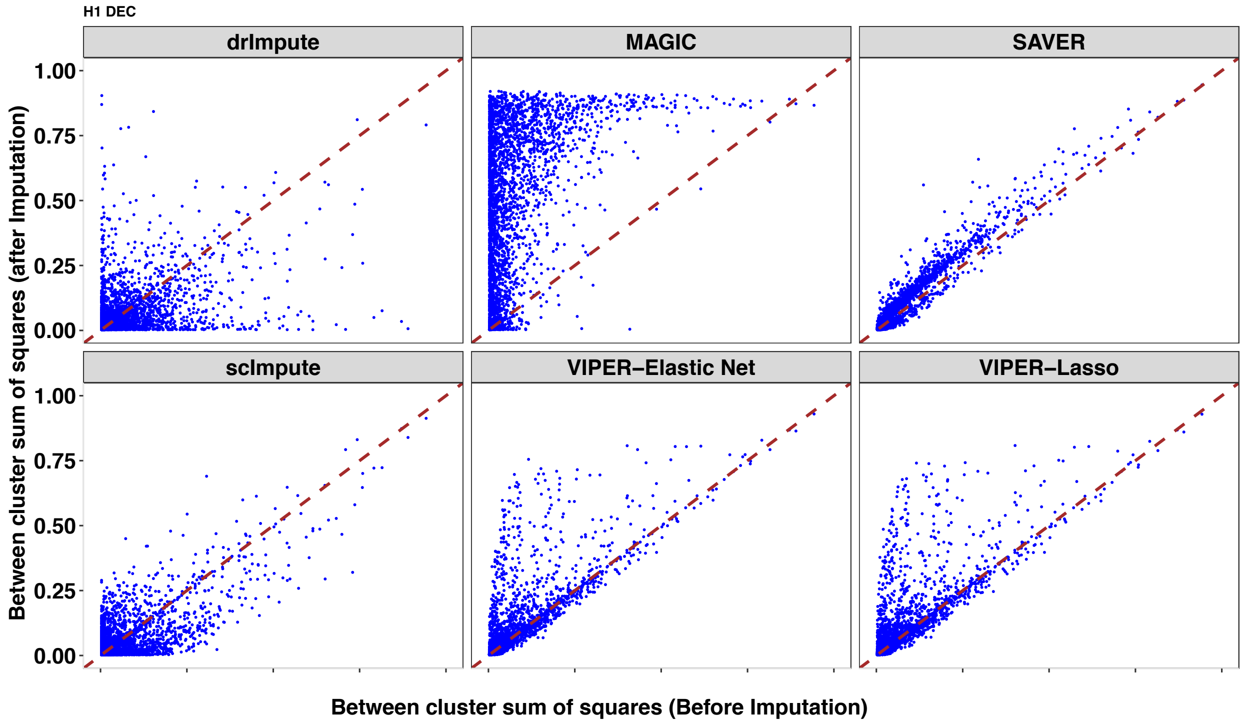

Fig S18-20: Comparison of between cluster sum of squares of the original value before imputation (x-axis) and sum of squares of the imputed value after imputation (y-axis) for three pairs of cell types in the Cell Type data. The three pairs of cell types include H1 and DEC in **S18**, EC and HFF in **S19**, and NPC and TB in **S20**. Between cluster sum of squares are computed for imputed data by different imputation methods that include DrImpute, MAGIC, SAVER, scImpute, VIPER with elastic net selection, and VIPER with lasso selection.

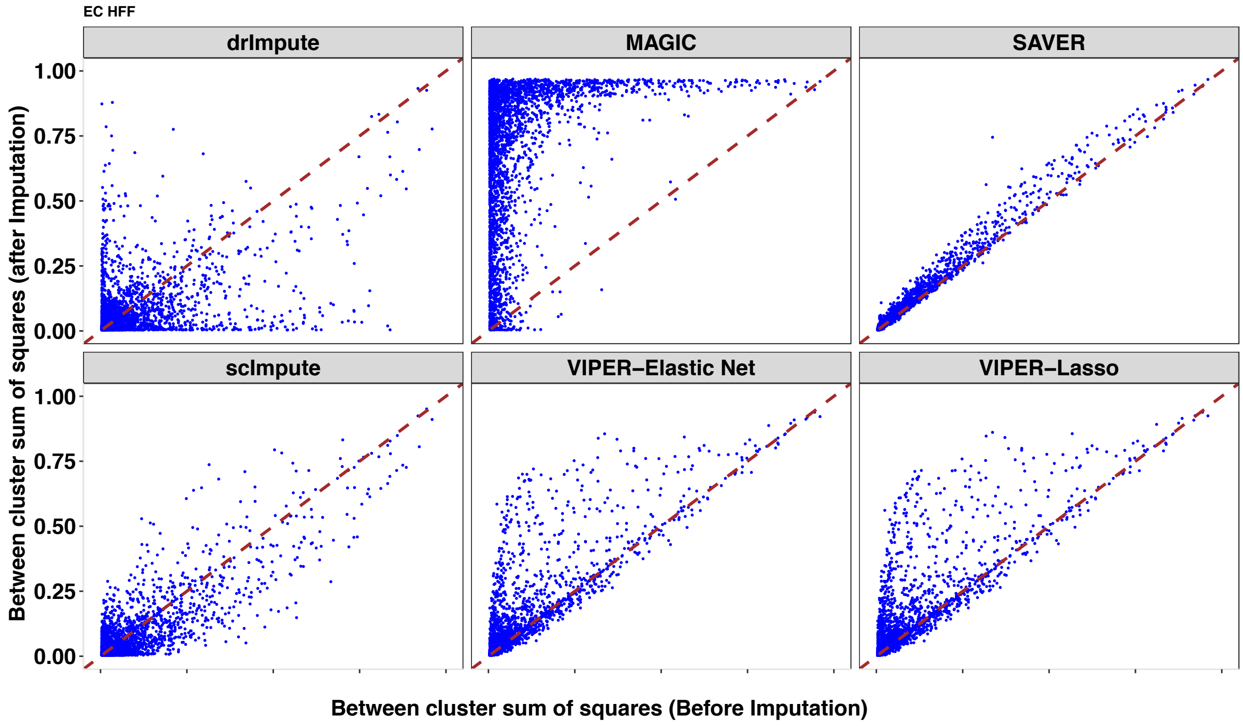

Fig S18-20: Comparison of between cluster sum of squares of the original value before imputation (x-axis) and sum of squares of the imputed value after imputation (y-axis) for three pairs of cell types in the Cell Type data. The three pairs of cell types include H1 and DEC in **S18**, EC and HFF in **S19**, and NPC and TB in **S20**. Between cluster sum of squares are computed for imputed data by different imputation methods that include DrImpute, MAGIC, SAVER, scImpute, VIPER with elastic net selection, and VIPER with lasso selection.

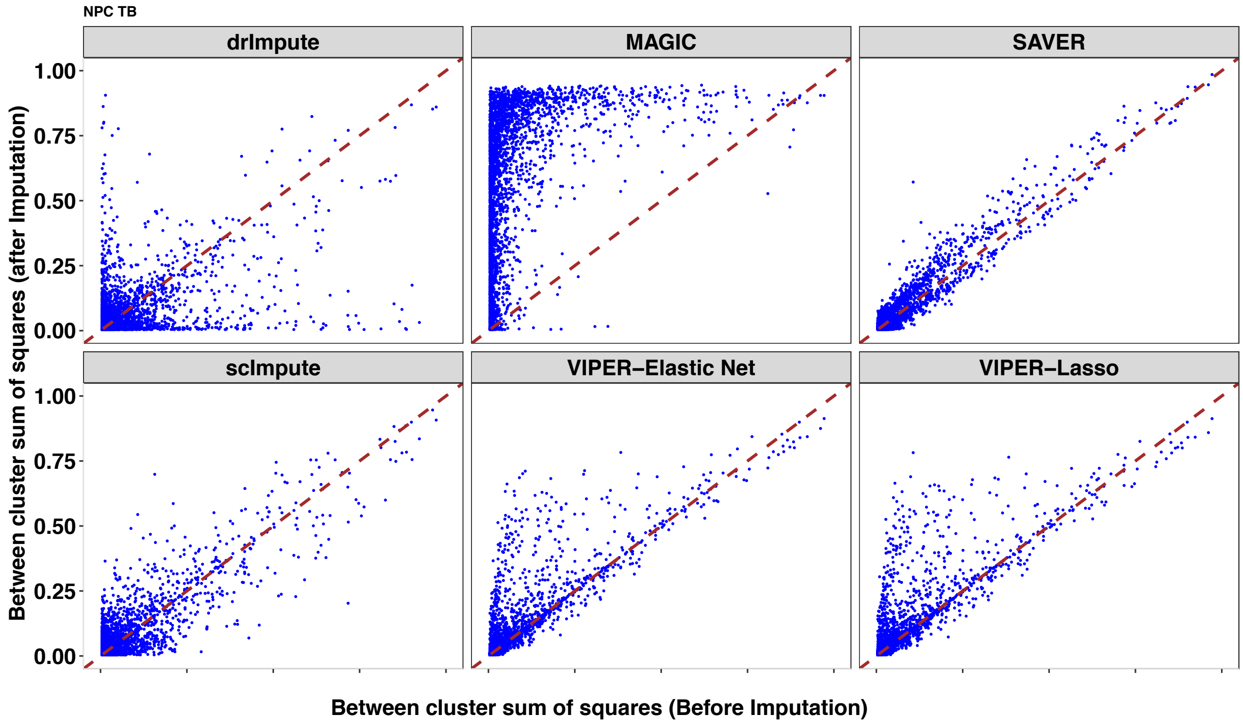

Supplementary Figures S18-20: Comparison of between cluster sum of squares of the original value before imputation (x-axis) and sum of squares of the imputed value after imputation (y-axis) for three pairs of cell types in the Cell Type data. The three pairs of cell types include H1 and DEC in **S18**, EC and HFF in **S19**, and NPC and TB in **S20**. Between cluster sum of squares are computed for imputed data by different imputation methods that include DrImpute, MAGIC, SAVER, scImpute, VIPER with elastic net selection, and VIPER with lasso selection.

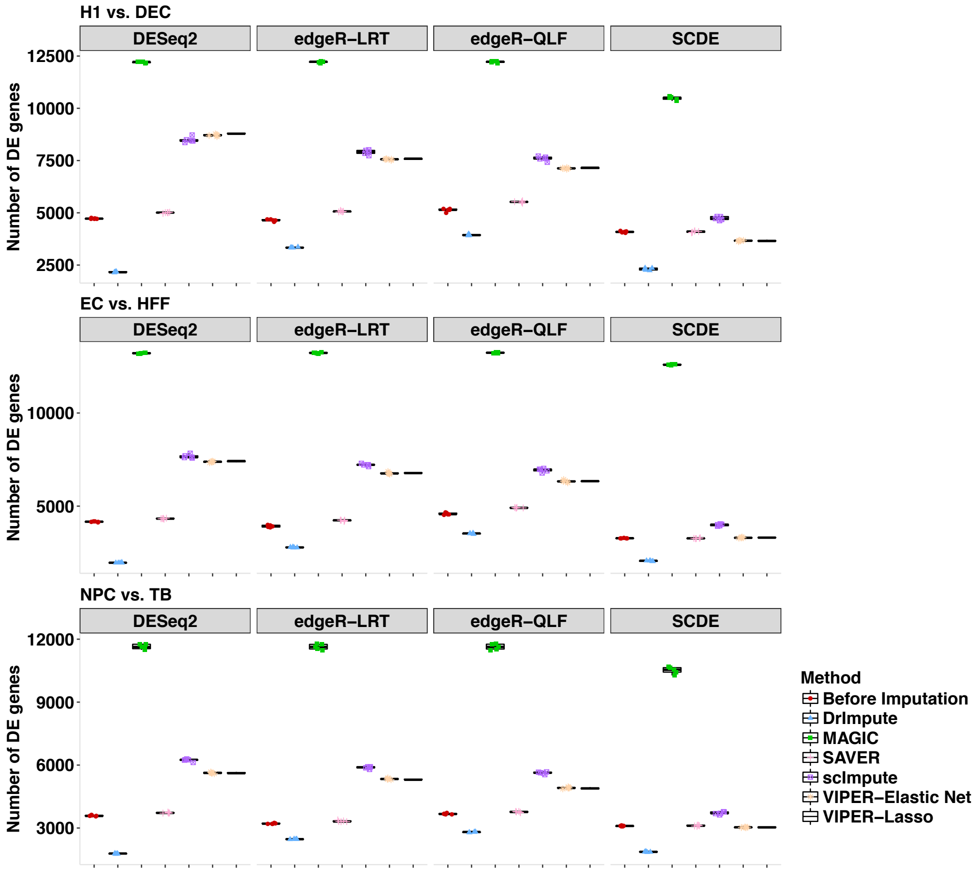

Fig S21: Number of differentially expressed genes detected in unimputed or imputed data. Differentially expression tools that include DESeq2 (first column), edgeR-LRT (second column), edgeR-QLF (third column) or SCDE (fourth column) are applied to detect genes that are differentially expressed between two cell subpopulations in the Cell Type data. The compared cell subpopulations include H1 vs DEC (first row), EC vs HFF (second row), or NPC vs TB (third row). In each comparison, the expression data are split randomly into two subsets. Imputation and differential expression analysis methods are applied to each data subset separately. The total number of differentially expressed genes based on a nominal p value cutoff of 0.01 are computed across 10 random data splits for each imputation method. Methods for comparison include DrImpute (blue), MAGIC (green), SAVER (pink), scImpute (purple), VIPER with elastic net selection (peach), and VIPER with lasso selection (dark blue).

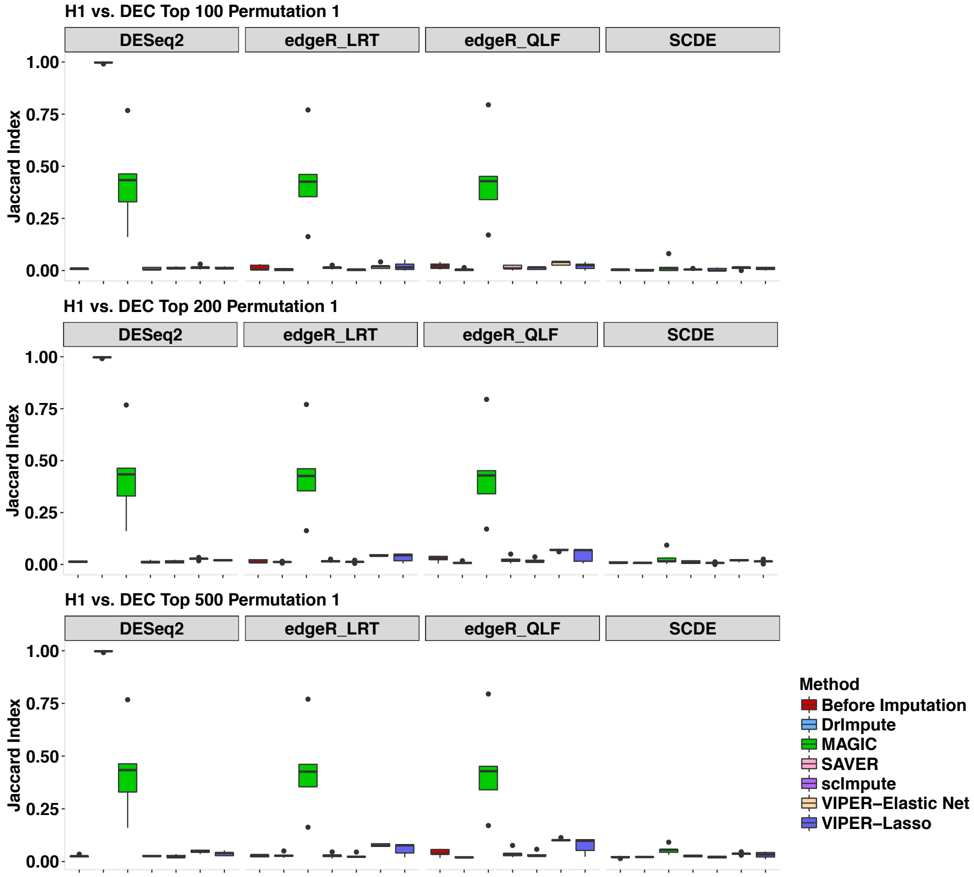

Fig S22-23: VIPER imputation facilitates the robust detection of differentially expressed genes without introducing bias to randomly split subsets. We first permuted the phenotype labels of H1 vs. DEC and then generate 10 randomly split copies. Then we applied imputation and differential expression analysis methods to each split separately. The Jaccard Indexes between the top 100, 200 and 500 differentially expressed genes detected from DESeq2 (first column), edgeR-LRT (second column), edgeR-QLF (third column) or SCDE (fourth column) are reported for two permutations (permutation #1 in **S22**, permutation #2 in **S23**). Imputation methods for comparison include DrImpute (blue), MAGIC (green), SAVER (pink), scImpute (purple), VIPER with elastic net selection (peach), and VIPER with lasso selection (dark blue).

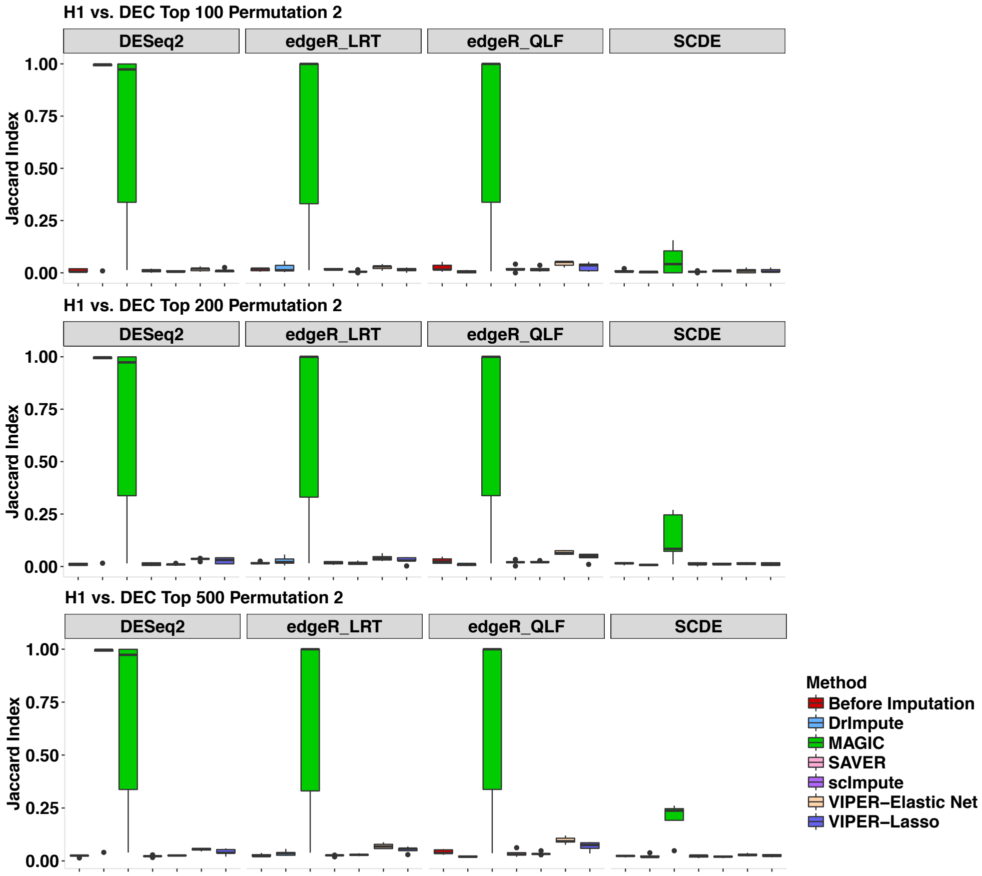

Fig S22-23: VIPER imputation facilitates the robust detection of differentially expressed genes without introducing bias to randomly split subsets. We first permuted the phenotype labels of H1 vs. DEC and then generate 10 randomly split copies. Then we applied imputation and differential expression analysis methods to each split separately. The Jaccard Indexes between the top 100, 200 and 500 differentially expressed genes detected from DESeq2 (first column), edgeR-LRT (second column), edgeR-QLF (third column) or SCDE (fourth column) are reported for two permutations (permutation #1 in **S22**, permutation #2 in **S23**). Imputation methods for comparison include DrImpute (blue), MAGIC (green), SAVER (pink), scImpute (purple), VIPER with elastic net selection (peach), and VIPER with lasso selection (dark blue).

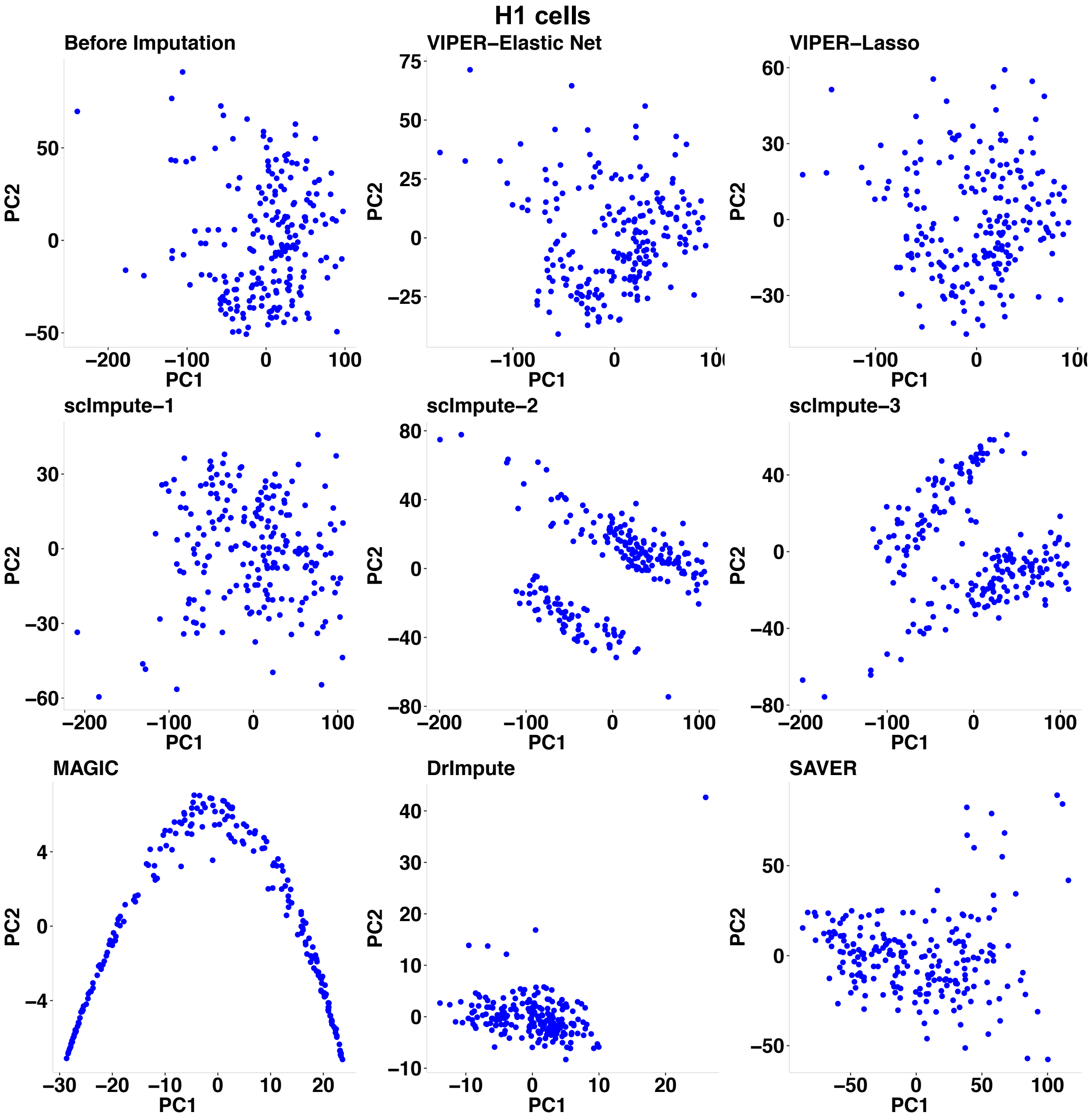

Fig S24-S25: Clustering results on the raw data or imputed data from different imputation methods for either H1 cells (**S24**) or NPC cells (**S25**). Cells are shown on PCA plots based on principal components 1 and 2. Imputation methods for comparison include DrImpute, MAGIC, SAVER, scImpute, VIPER with elastic net selection, and VIPER with lasso selection. scImpute requires pre-specifying the number of cell subpopulations before clusters and we set this number to be 1, 2 or 3.

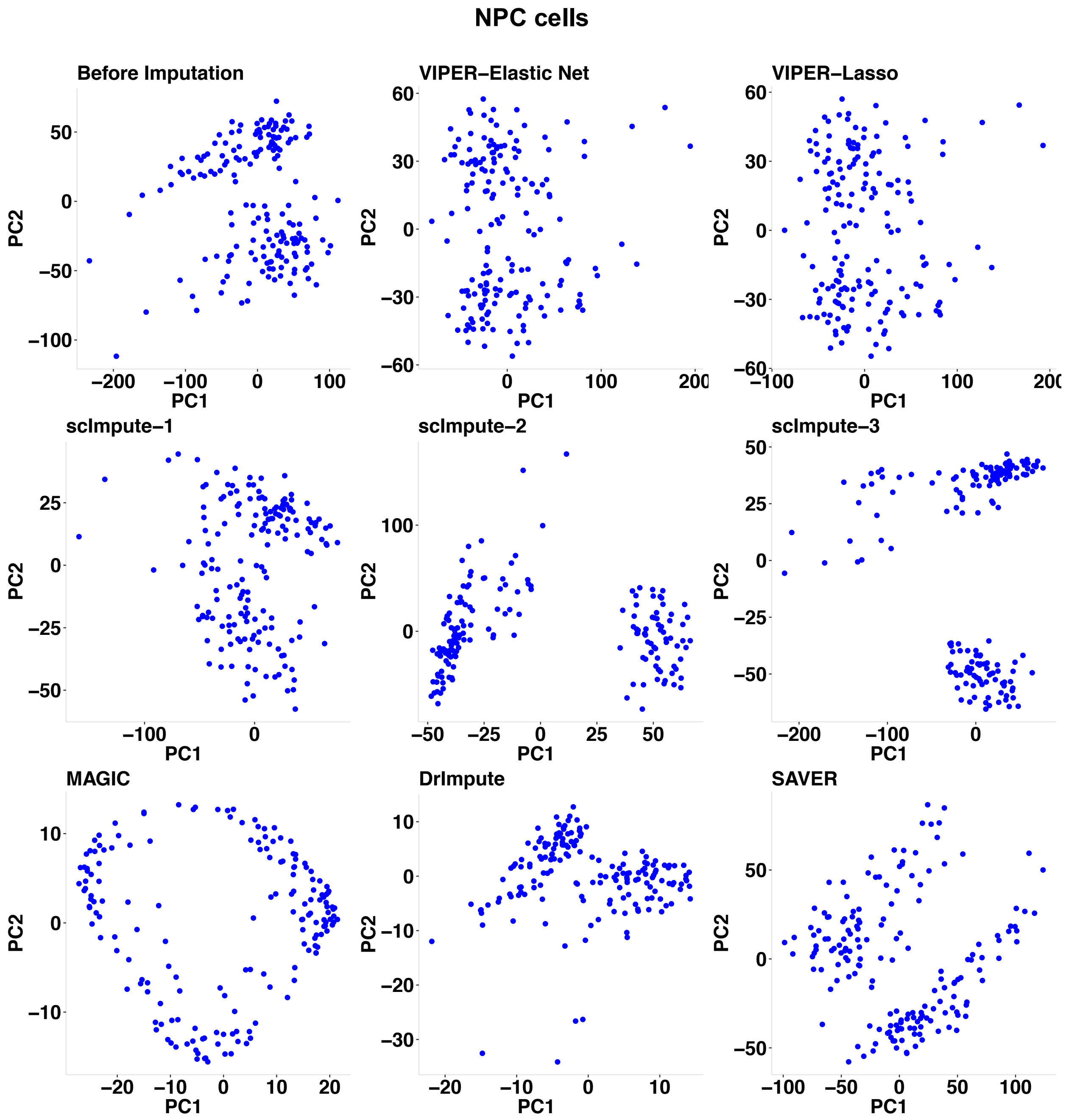

Fig S24-S25: Clustering results on the raw data or imputed data from different imputation methods for either H1 cells (**S24**) or NPC cells (**S25**). Cells are shown on PCA plots based on principal components 1 and 2. Imputation methods for comparison include DrImpute, MAGIC, SAVER, scImpute, VIPER with elastic net selection, and VIPER with lasso selection. scImpute requires pre-specifying the number of cell subpopulations before clusters and we set this number to be 1, 2 or 3.

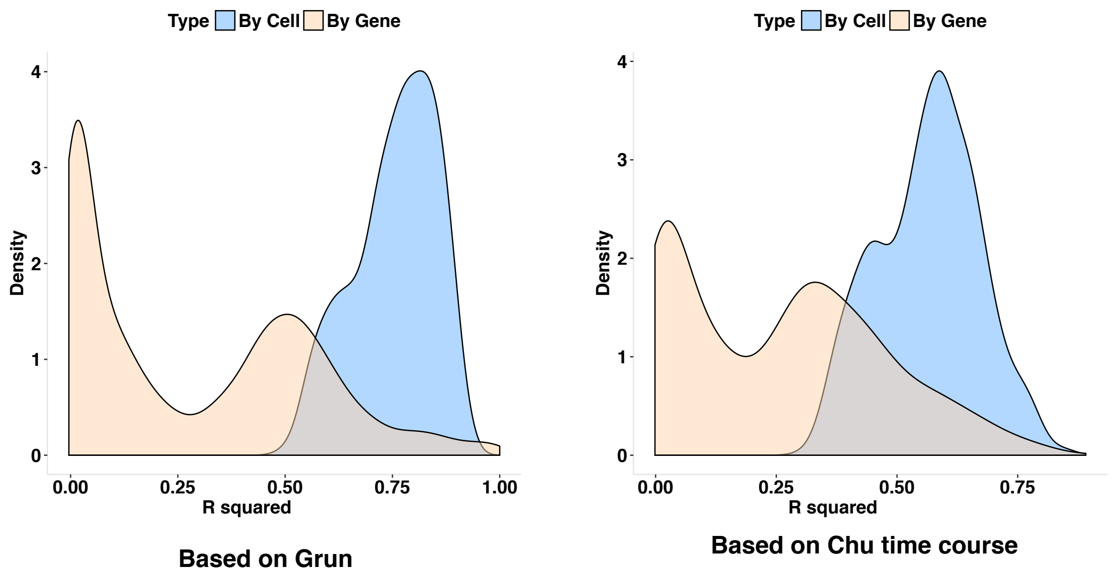

Fig S26: Using neighborhood cells for imputation is more accurate than using neighborhood genes for imputation in both the Cell Type data (left panel) and Time Course data (right panel). A standard lasso regression is applied to either use cells to predict the cell of interest (light blue) or use genes to predict the gene of interest (light orange). In-sample R^2 across genes (light blue) or across cells (light orange) are plotted.
