# Supplementary material for: VIPER: variability-preserving imputation for accurate gene expression recovery in single-cell RNA sequencing studies
Source: Genome Biol. 2018 Nov 12;19:196. doi: 10.1186/s13059-018-1575-1 (PMC6233584; doi:10.1186/s13059-018-1575-1)
Supplement: Supplementary file 3 — Tables S1 and S2. Percentage of zero values in the downsampling experiments and computating time comparisons. (PDF 150 kb) [file 13059_2018_1575_MOESM3_ESM.pdf]

Downsampling Rate	Independent	Grun	Cell Type	Time Course	Shalek
0.95	Y	30.29	73.13	62.77	50.15
0.9	Y	30.41	72.58	62.29	49.22
0.8	Y	28.62	70.32	59.86	46.34
0.7	Y	24.39	65.23	54.07	40.32
0.6	Y	16.18	52.29	41.08	28.16
0.5	Y	9.49	37.13	27.17	17.44
0.95	N	8.5	45.59	36.73	30.52
0.9	N	8.52	43.7	35.59	29.38
0.8	N	8.39	41	33.95	27.84
0.7	N	8.19	38	31.87	25.97
0.6	N	7.84	34.37	29.51	23.99
0.5	N	7.68	32.62	28.43	22.95

Table S1: Percentage of zero values that are due to down-sampling in the down-sampling experiment based on four real data sets. In the experiment, down-sampling rate ranges from 0.5 to 0.95 and the dropout rate is either dependent (N) or independent (Y) of the expression level. The remaining percentage of zero values that are not due to down-sampling are due to dropout events.

dataset	dimensionality ($n \times p$)	Proportion of zeros	DrImpute	MAGIC	SAVER	scImpute	VIPER-Elastic Net	VIPER-Lasso
Grun	$251 \times 12,184$	39%	0.004	0.002	7.25	0.05	0.34	0.33
Cell Type	$1,018 \times 13,829$	31%	0.04	0.009	30.09	1.67	4.27	3.82
Time Course	$758 \times 13,059$	34%	0.03	0.007	21.37	0.51	5.42	5.15
Shalek	$1,053 \times 16,702$	48%	0.05	0.013	46.34	0.20	10.6	10.6

Table S2: Computing time in hours for different imputation methods in the four real datasets. Number of cells (n), number of genes (p) as well as the proportion of zero entries in each data are listed. Computing time is evaluated on an Intel E5-2680v4 2.4GHz CPU. Methods for comparison include DrImpute, MAGIC, SAVER, scImpute, VIPER with elastic net selection, and VIPER with lasso selection.
